# Advanced microscopy resolves dynamic localization patterns of stress-induced mitogen-activated protein kinase (SIMK) during alfalfa root hair interactions with *Ensifer meliloti*

**DOI:** 10.1093/jxb/erad111

**Published:** 2023-03-23

**Authors:** Kateřina Hlaváčková, Olga Šamajová, Miroslava Hrbáčková, Jozef Šamaj, Miroslav Ovečka

**Affiliations:** Department of Biotechnology, Faculty of Science, Palacký University Olomouc, Olomouc, Czech Republic; Department of Biotechnology, Faculty of Science, Palacký University Olomouc, Olomouc, Czech Republic; Department of Biotechnology, Faculty of Science, Palacký University Olomouc, Olomouc, Czech Republic; Department of Biotechnology, Faculty of Science, Palacký University Olomouc, Olomouc, Czech Republic; Department of Biotechnology, Faculty of Science, Palacký University Olomouc, Olomouc, Czech Republic; University of Tasmania Australia

**Keywords:** Alfalfa, *Ensifer meliloti*, infection pocket, immunolocalization, infection thread, light-sheet fluorescence microscopy, MAPKs, root hairs, SIMK, subcellular localization

## Abstract

Leguminous plants have established mutualistic endosymbiotic interactions with nitrogen-fixing rhizobia to secure nitrogen sources in root nodules. Before nodule formation, the development of early symbiotic structures is essential for rhizobia docking, internalization, targeted delivery, and intracellular accommodation. We recently reported that overexpression of stress-induced mitogen-activated protein kinase (SIMK) in alfalfa affects root hair, nodule, and shoot formation, raising the question of how SIMK modulates these processes. In particular, detailed subcellular spatial distribution, activation, and developmental relocation of SIMK during early stages of alfalfa nodulation remain unclear. Here, we characterized SIMK distribution in *Ensifer meliloti*-infected root hairs using live-cell imaging and immunolocalization, employing alfalfa stable transgenic lines with genetically manipulated SIMK abundance and kinase activity. In the *SIMKK-RNAi* line, showing down-regulation of *SIMKK* and *SIMK*, we found considerably decreased accumulation of phosphorylated SIMK around infection pockets and infection threads. However, this was strongly increased in the GFP-SIMK line, constitutively overexpressing green fluorescent protein (GFP)-tagged SIMK. Thus, genetically manipulated SIMK modulates root hair capacity to form infection pockets and infection threads. Advanced light-sheet fluorescence microscopy on intact plants allowed non-invasive imaging of spatiotemporal interactions between root hairs and symbiotic *E. meliloti*, while immunofluorescence detection confirmed that SIMK was activated in these locations. Our results shed new light on SIMK spatiotemporal participation in early interactions between alfalfa and *E. meliloti*, and its internalization into root hairs, showing that local accumulation of active SIMK modulates early nodulation in alfalfa.

## Teaser

Genetic down-regulation, overexpression, and advanced microscopy revealed that dynamic relocation and activation of stress-induced mitogen-activated protein kinase (SIMK) modulate alfalfa root hair capacity to interact with *Ensifer meliloti*.

## Introduction

Nitrogen shortage in the soil is one of the major factors restricting the growth and productivity of plants, including crops. To overcome or alleviate this limitation, *Medicago sativa* L. (alfalfa), a legume crop of high agronomic and ecological importance, is able to acquire nitrogen by symbiotic hosting of nitrogen-fixing rhizobia in *de novo* formed specialized organs, root nodules (Checcucci *et al.*, 2016; Wang *et al.*, 2018). Root nodules provide rhizobia with favourable conditions to convert atmospheric dinitrogen (N_2_) into ammonia (NH_3_) in the process of biological nitrogen fixation. Rhizobia export nitrogen-rich compounds to the host plant in exchange for carbohydrates that are utilized by rhizobia as a source of carbon and energy (White *et al.*, 2007; Oldroyd *et al.*, 2011). The legume–rhizobium symbiosis is established through a complex developmental process that starts with the exchange of signalling molecules between the host and symbiont and the activation of signal transduction pathways, triggering the nodulation programme in the host legume plant (Oldroyd, 2013; Yang *et al.*, 2022). Flavonoids secreted by legume roots attract rhizobia and stimulate them to produce nodulation factors (NFs) (Dénarié and Cullimore, 1993; Clúa *et al.*, 2018; Kidaj *et al.*, 2020). Perception of the correct NF structure by compatible receptors in legume root cells initiates early nodulation steps and prepares the host legume plant for symbiotic infection by invading rhizobia (Gage, 2004; Timmers, 2008; Roy *et al.*, 2020).

Nodule formation requires two separate, but spatially and temporally highly coordinated processes: rhizobial infection of root hairs and nodule organogenesis in the root cortex (Oldroyd and Downie, 2008; Ibáñez *et al.*, 2017). Before nodules arise as newly formed functional and nitrogen-fixing root lateral organs, rhizobia must travel from the root surface towards the target cells in the inner root tissue. In the initial stage, rhizobia attach to the growing root hair tips and are trapped in the root hair curls, creating enclosed chambers known as infection pockets (Fournier *et al*., 2015; Rae *et al.*, 2021). Within the infection pockets, rhizobia divide and form colonies referred to as infection foci from which infection threads (ITs) entering root hairs are initiated by inverted tip growth. These plant-made tube-like membrane channels are filled with rhizobia, grow down towards the base of infected root hair, and branch out by growing through the root cortex. Eventually, the inward-growing IT and the outward-growing root nodule primordium meet inside the root tissue (Fournier *et al*., 2008; Rashid *et al.*, 2015). When ITs reach the nodule primordium, rhizobia are released into the cytoplasm of host cells by endocytosis, become surrounded by plant-derived peribacteroid membrane, and differentiate into bacteroids that are responsible for nitrogen fixation by the activity of the nitrogenase enzymatic complex (Terpolilli *et al.*, 2012; Poole *et al.*, 2018).

Within a complex signalling network controlling the nodulation process, mitogen-activated protein kinases (MAPKs) become activated early after rhizobial infection (Lopez-Gomez *et al*., 2012). MAPK cascades represent conserved and universal signalling hubs transducing external stimuli into target substrates by a sequential action of three protein kinases, MAPK kinase kinase (MAPKKK), MAPK kinase (MAPKK), and MAPK. Plant MAPKs can be activated by a variety of biotic and abiotic stress stimuli. During signal transduction, MAPKKK reversibly activates its downstream MAPKK, which phosphorylates and activates MAPK by dual phosphorylation of threonine (T) and tyrosine (Y) residues of the T-X-Y motif (Pitzschke, 2015; Xu and Zhang, 2015; Zhang and Zhang, 2022). Activated MAPKs phosphorylate and regulate many diverse substrates such as transcription factors, enzymes, cytoskeletal proteins, or other kinases. Signalling through MAPK modules regulates a broad range of cellular and developmental processes as well as pathogenic or beneficial biotic interactions (Rasmussen *et al.*, 2012; Šamajová *et al.*, 2013; Smékalová *et al.*, 2014; Komis *et al.*, 2018; Sun and Zhang, 2022).

Although MAPK-mediated phosphorylation cascades represent an essential component of plant cell signalling, still relatively little is known about MAPKs in legume crops. In alfalfa, stress-induced MAPK (SIMK) is activated by biotic and abiotic stimuli such as fungal elicitors and salt stress, respectively (Munnik *et al.*, 1999; Cardinale *et al.*, 2000, 2002). Activation analyses and yeast two-hybrid screening identified SIMK kinase (SIMKK) as a SIMK-specific activator. SIMKK directly activates SIMK in response to salt stress (Kiegerl *et al.*, 2000; Cardinale *et al.*, 2002), and localization studies at the subcellular level revealed substantial relocation of both SIMKK and SIMK from nuclei to the cytoplasmic spot-like compartments upon salt stress (Ovečka *et al*., 2014). In addition, activated SIMK relocates from nuclei to the tips of growing root hairs and, together with the dynamic actin cytoskeleton, regulates alfalfa root hair tip growth (Šamaj *et al.*, 2002, 2003). Most importantly, we have recently addressed SIMK positive role in alfalfa nodulation and development through its genetic manipulations. For this functional assessment, a transgenic *SIMKK-RNAi* line with a strong down-regulation of SIMK production and activity and a transgenic GFP-SIMK line constitutively overexpressing green fluoescent protein (GFP)-tagged and activated SIMK were utilized. SIMK overexpression promoted root hair growth, ITs, and nodule clustering, as well as positively affecting agronomical traits such as shoot biomass production, suggesting the biotechnological potential of this kinase (Hrbáčková *et al.*, 2021). However, the functional and spatiotemporal mode of SIMK participation at early nodulation stages remains unknown.

In this study, live-cell imaging using light-sheet fluorescence microscopy (LSFM) supplemented with quantitative microscopy employing alfalfa-adapted immunolabelling techniques revealed SIMK-specific subcellular localization and activation at the early stages of the alfalfa–*Ensifer meliloti* symbiotic interaction process. Advanced LSFM was essential to secure unique imaging conditions for alfalfa–*E. meliloti* interaction that are highly compromised in conventional microscopy approaches. Considering the robustness of alfalfa roots, the classical slide–coverslip sandwich mounting approach would undesirably restrict the root environment of mounted plants during long-term recordings. In LSFM, samples were inserted vertically into the observation chamber and, unlike conventional microscopy systems, plants were maintained in a natural gravity-oriented manner during long-term imaging. Moreover, regions of interest on imaged roots can be freely and precisely positioned (including rotational positioning) in front of the detection objective. Structural integrity and tip growth of root hairs are very sensitive to imaging conditions, including mechanical manipulation and high illumination intensity typically used in conventional microscopy. In LSFM, very low levels of excitation energy absorbed by samples, and a voluminous chamber providing enough space for root and root hair growth, provided conditions close to natural ones. This advanced LSFM microscopy revealed that SIMK in the root hair accumulated in the docking site where *E. meliloti* was attached, entrapped, and internalized. We correlated SIMK subcellular localization patterns in root hairs at *E. meliloti* internalization sites in two contrasting alfalfa genotypes, stably overexpressing GFP-tagged SIMK or down-regulating the SIMK level by means of *SIMKK* RNAi technology, respectively. SIMK genetic manipulation, the mode of activation, and the localization pattern indicate that the effectiveness of early nodulation steps in alfalfa is modulated by precise spatiotemporal SIMK activation.

## Materials and methods

### Plant material

Alfalfa wild-type plants [cv. Regen-SY (RSY)] and RSY plants carrying either the *35S::GFP:SIMK* construct (producing GFP–SIMK fusion protein; Hrbáčková *et al.*, 2021) or *SIMKK-RNAi* in the pHellsgate12 plasmid driven under the *35S* promoter (obtained from CSIRO Plant Industry, Australia) were obtained by regeneration *in vitro* through somatic embryogenesis from leaf explants as previously described (Samac and Austin-Phillips, 2006; Bekešová *et al*., 2015; Hrbáčková *et al.*, 2021). From somatic embryos, regenerated alfalfa plants of RSY, the transgenic *SIMKK-RNAi* line (showing strong down-regulation of *SIMKK* and *SIMK* transcripts and SIMK protein), and the transgenic GFP-SIMK line with up-regulated *SIMK* transcript and enhanced SIMK activity (Hrbáčková *et al.*, 2021) were transferred to nitrogen-free Fåhreus medium [macronutrients: 0.5 mM MgSO_4_·7H_2_O, 0.7 mM KH_2_PO_4_, 0.8 mM Na_2_HPO_4_·2H_2_O, 100 µM Fe-EDTA; micronutrients: 0.1 µg l^–1^ MnSO_4_·H_2_O, CuSO_4_·5H_2_O, ZnSO_4_·H_2_O, H_3_BO_3_, Na_2_MoO_4_·2H_2_O; pH 6.5; 1 mM CaCl_2_ added after autoclaving] (FAH-N_2_; Fåhreus, 1957) for inoculation with rhizobia.

### Plant inoculation with *E. meliloti*

For live-cell imaging, regenerated plantlets of the transgenic GFP-SIMK line, 3–4 day-old and ~1.5 cm long, growing on FAH-N_2_ medium containing 13 g l^–1^ micro agar (10 plantlets per plate), were inoculated with *E. meliloti* (strain Sm2011) producing monomeric red fluorescent protein (mRFP) with OD_600_=0.5 (2.50e+008 cells ml^–1^) and imaged 3–4 days post-inoculation (dpi). Plant inoculation was performed by application of bacterial suspension (2 ml per 10 plantlets on the plate) directly to the root surface on the agar in Petri plates. For quantitative analyses of IT numbers, 18-day-old plants of alfalfa RSY and transgenic *SIMKK-RNAi* and GFP-SIMK lines originating from somatic embryos and growing on FAH-N_2_ medium (three plants per plate) were inoculated with *E. meliloti* wild type (strain Sm2011) with OD_600_=0.5. In total, 2 ml of rhizobial suspension was applied to the root system surface directly on plates, followed by vertical cultivation of inoculated plants in an environmental chamber at 21 °C, 70% humidity, and 16 h/8 h light/dark cycle with covered root systems.

### Sample preparation for live-cell imaging and LSFM

Plantlets of the transgenic GFP-SIMK line were used for live-cell imaging to observe *in vivo* localization and dynamics of GFP-tagged SIMK during the early stages of nodulation. Samples for LSFM imaging were prepared according to Ovečka *et al*. (2015) and Valuchova *et al.* (2020). A fluorinated ethylene propylene (FEP) tube with an inner diameter of 4.2 mm and outer diameter of 4.6 mm was connected to the glass capillary (inner diameter of 2.15 mm and outer diameter of 4.0 mm) using a hot glue gun (Supplementary Fig. S1A). The inoculated plantlet was gently inserted into the FEP tube with tweezers, and medium (FAH-N_2_ medium, pH 6.5) with 1% (w/v) low melting point agarose (Sigma Aldrich) containing fiducial markers (fluorescent beads of 1 µm in diameter, typically used for multiangular acquisition allowing post-acquisition bead-based registration of microscopy images and their alignment to achieve efficient and sample-independent 3D rendering) was slowly added from the bottom into the FEP tube. Under these conditions, the plant root was embedded in the solidified block of the culture medium inside the FEP tube while the green upper part of the plant was exposed to air (Supplementary Fig. S1A, C). The glass capillary connected to the FEP tube containing the embedded sample was fixed into the metal holder (Supplementary Fig. S1B) and directly placed into a pre-tempered (22 °C) LSFM observation chamber filled with liquid FAH-N_2_ medium. After sample stabilization, imaging was performed using the light-sheet Z.1 fluorescence microscope (Carl Zeiss, Germany) equipped with a Plan-Apochromat ×10/0.5 NA detection objective and two LSFM ×10/0.2 NA illumination objectives (Carl Zeiss, Germany). Rhizobia-infected roots were imaged using dual-side light-sheet illumination with excitation laser lines 488 nm for GFP (beam splitter LP 560 and emission filter BP 505–545) and 561 nm for RFP (beam splitter LP 560 and emission filter BP 575–615). Images were acquired with the PCO. Edge sCMOS cameras (PCO AG, Germany) with an exposure time of 50 ms and an imaging frequency of every 2 min in *z*-stack mode for 80–120 min. The scaling of acquired images in *x*, *y*, and *z* dimensions was 0.466 µm×0.466 µm×1.497 µm, and light-sheet thickness was set to the optimal value.

### Fixation of alfalfa root samples

For whole-mount immunofluorescence labelling, ~1.5 cm long root segments including the root apex were excised from primary and lateral roots of 18-day-old alfalfa RSY, *SIMKK-RNAi*, and GFP–SIMK plants co-cultivated with *E. meliloti* and fixed in freshly prepared fixative solution [2% (v/v) paraformaldehyde, 0.2% (v/v) glutaraldehyde, 0.3% (v/v) Tween-20, 0.3% (v/v) Triton X-100, and 10% (v/v) DMSO in half-strength MTSB (50 mM PIPES, 5 mM MgSO_4_·7H_2_O, 5 mM EGTA, pH 6.9)] (Tichá *et al.*, 2020). Sampling was done at 3–7 dpi with *E. meliloti* when infection pockets (3–4 dpi) and growing ITs (6–7 dpi) were clearly detectable inside rhizobia-infected root hairs after microscopic observation.

### Immunolabelling of SIMK and activated MAPKs in symbiotically infected root hairs

SIMK subcellular localization at early symbiotic stages was performed by immunofluorescence labelling on fixed root samples of alfalfa RSY, *SIMKK-RNAi*, and GFP-SIMK lines co-cultivated with *E. meliloti* using a SIMK-specific antibody. To check out the activation state of SIMK in analysed early stages of nodulation, an activated pool of MAPKs was immunodetected using a phospho-specific antibody (anti-phospho-p44/42, Cell Signalling, the Netherlands). Root samples were simultaneously double-immunolabelled with rabbit anti-AtMPK6 (SIMK-specific) primary antibody (Sigma, Life Science, USA) at 1:750 dilution in 2.5% (w/v) BSA in phosphate-buffered saline [PBS; 140 mM NaCl, 2.7 mM KCl, 6.5 mM Na_2_HPO_4_·2H_2_O, 1.5 mM KH_2_PO_4_, pH 7.3] for SIMK localization and with mouse anti-phospho-p44/42 primary antibody at 1:400 dilution in 2.5% (w/v) BSA in PBS to visualize activated MAPKs. Vacuum infiltration was used (3 × 5 min) to improve antibody penetration, followed by overnight incubation at 4 °C. Samples were then sequentially incubated with appropriate Alexa Fluor-conjugated secondary antibodies. First, Alexa Fluor 647 rabbit anti-mouse secondary antibody (Abcam) diluted 1:500 in 2.5% (w/v) BSA in PBS was used for 2 h incubation at 37 °C. Samples were extensively washed in PBS (5 × 10 min), blocked in 5% (w/v) BSA in PBS for 20 min at room temperature, and incubated with Alexa Fluor 555 goat anti-rabbit secondary antibody (Abcam) by keeping the same dilution and incubation conditions. In the last step of the immunolabelling procedure, nuclei and *E. meliloti* were visualized with 1 µg ml^–1^ DAPI diluted 1:1000 in PBS. Samples were stained at room temperature in the darkness for 15 min.

### FM4-64 staining

The fixable variant of the styryl dye FM4-64FX (Invitrogen, USA) was used for *in situ* visualization of plasma membranes in alfalfa root cells treated with *E. meliloti*. Roots of 18-day-old plants were labelled in liquid FAH-N_2_ medium (pH 6.5) containing FM4-64FX at a final concentration of 4 μM in 5 ml Eppendorf tubes on ice for 20 min. The excess dye was quickly washed out with liquid FAH-N_2_ medium, and primary and lateral roots were cut into 1.5 cm long segments including the root tip and immediately fixed in freshly prepared fixative solution [2% (v/v) paraformaldehyde, 0.2% (v/v) glutaraldehyde, 0.3% (v/v) Tween-20, 0.3% (v/v) Triton X-100, and 10% (v/v) DMSO in half-strength MTSB] (Tichá *et al*., 2020). Fixed root segments were used for immunolabelling as described above. For SIMK immunostaining in FM4-64FX-labelled samples, rabbit SIMK-specific primary and Alexa Fluor 647 goat anti-rabbit secondary antibodies were used.

### Confocal laser scanning microscopy (CLSM) and Airyscan CLSM

Root samples immunolabelled for SIMK and activated MAPKs were mounted in antifade mounting medium [0.1% (w/v) paraphenylenediamine in 90% (v/v) glycerol buffered with 10% (v/v) PBS at pH 8.2–8.6] to protect samples from photo-bleaching, and used for microscopy. Imaging of immunolabelled SIMK and activated MAPKs was performed with the Zeiss LSM 710 platform (Carl Zeiss, Germany) equipped with Plan-Apochromat ×40/1.4 Oil DIC M27 and Plan-Apochromat ×63/1.4 Oil DIC M27 objectives. Samples were imaged with excitation laser lines at 405 nm for DAPI, 488 nm for detection of GFP, 561 nm for Alexa Fluor 555 to visualize SIMK, and 631 nm for Alexa Fluor 647 to detect activated MAPKs. Microscopic analysis of immunostained SIMK and FM4-64FX-visualized membranes in rhizobia-infected root hairs was performed with a Zeiss LSM 880 Airyscan equipped with 32 GaAsP detectors (Carl Zeiss, Germany) using a Plan-Apochromat ×63/1.4 Oil DIC M27 objective. Samples were imaged with excitation laser lines at 561 nm for FM4-64FX and 631 nm for Alexa Fluor 647.

### Quantification of ITs

For quantitative evaluation of IT formation, 18-day-old plants of RSY, *SIMKK-RNAi*, and GFP-SIMK lines, originating from somatic embryos, were inoculated with *E. meliloti* wild type applied to roots directly on the agar surface. Inoculated plants were daily subjected to microscopic observations from 4 to 10 dpi. ITs were counted and the evaluation of IT number per the root system length was performed at 10 dpi with *E. meliloti* using an Axio Zoom.V16 Stereo microscope (Carl Zeiss, Germany).

### Image acquisition and processing

The image acquisition, post-processing, semi-quantitative profile measurements, quantitative co-localization analysis, maximum intensity projections from individual *z*-stacks, subset creation of all fluorescence images, and 3D modelling were performed using Zeiss ZEN software (Black and Blue versions, Carl Zeiss, Germany). Data obtained by LSFM imaging were subjected to 3D rendering. A subset of selected *z*-stacks was created from the whole root volume to capture different stages of root nodulation. Data were imported to Arivis Vision4D 2.12.6 software (Arivis AG, Rostock, Germany), automatically converted to a *.sis file, displayed as 3D objects, and rendered in the maximum intensity mode. Animations and videos were prepared by clipping 3D models in *XZ* and *YZ* planes and using rotation and zooming tools in the 4D clipping panel by arranging keyframes. Although quantification of fluorescence intensities is not influenced by post-acquisition look-up table (LUT) intensity adjustments, all images used for semi-quantitative and quantitative analyses were acquired under the same imaging conditions. The same laser attenuation values for all laser lines were set prior to the acquisition, and the thickness of individual optical sections was optimized according to Nyquist criteria. The pinhole sizes for green (GFP), red (Alexa Fluor 555), and yellow (Alexa Fluor 647) channels were matched and the range of detection was appropriately adjusted to ensure separation of emission wavelengths and to prevent fluorescence spectral bleed-through. The brightness and contrast of all acquired images were uniformly adjusted and images exported from ZEN software were assembled into final figure plates using Microsoft PowerPoint.

### Semi-quantitative analysis of the fluorescence intensity distribution

Data obtained from LSFM live-cell imaging were semi-quantitatively evaluated by profile measurements to study the fluorescence intensity distribution of GFP-tagged SIMK in alfalfa root hairs and its association with *E. meliloti* at early symbiotic stages. GFP–SIMK mean fluorescence intensity from LSFM was quantitatively evaluated in specific regions of interest (ROIs), delineating nuclei and root hair tips of root hairs not interacting or interacting with *E. meliloti*. The measured area of nuclei and root hair tips of interacting root hairs for the quantification was selected manually with the drawing tool of the software, based on the size and shape of measured nuclei and infection pockets. In tips of non-interacting root hairs, a manually drawn ROI, representing the root hair tip, was created and the same mask was used for all non-growing root hair tips included in the measurement. Distribution of SIMK, GFP, and activated MAPKs around early invasion structures was determined on fixed and immunolabelled samples inside root hairs of alfalfa control and transgenic plants by semi-quantitative analysis and profile measurement of fluorescence intensities. Intensity profiles were quantified across infection pockets and ITs as indicated in appropriate images. These analyses were done using a profile or measure function of Zeiss ZEN 2011 software (Black version) from single confocal optical sections or maximum intensity projections.

### Quantitative co-localization analysis

The mode of co-localization of fluorescence signals was analysed on immunolabelled root samples of alfalfa control and transgenic plants co-cultivated with *E. meliloti*. Quantitative co-localization analysis between SIMK and activated MAPKs was conducted in particular ROIs at early symbiotic stages around infection pockets (3–4 dpi) and ITs (6–7 dpi). The measured area of infection pockets and ITs was selected manually with the drawing tool of the ZEN software, outlining the analysed region of early infection structures. The co-localization range was measured from single plane confocal sections. In total, three independent optical sections per infection pocket and IT were analysed using the co-localization tool of Zeiss ZEN 2014 software (Blue version). Background thresholds were automatically implemented by the iterative Costes approach (Costes *et al.*, 2004), and co-localization data were calculated from manually selected ROIs. Data were displayed in intensity-corrected scatterplot diagrams, the intensity correlation of co-localizing pixels was expressed by Pearson’s correlative coefficient, and results were graphically edited using Microsoft Excel.

### Statistical analysis

Statistical parameters of performed experiments, number of samples (*n*), and type of statistical test are included in the figure legends. Graphs were prepared in Microsoft Excel and finalized using PowerPoint software. Statistical significance (*P*<0.05) was determined using the Statistica 13.4.0 software (TIBCO Software Inc., Palo Alto, CA, USA) by one-way ANOVA with post-hoc Tukey HSD test (*P*<0.05).

## Results

### SIMK distribution in alfalfa growing root hairs

**Fig. 1.**
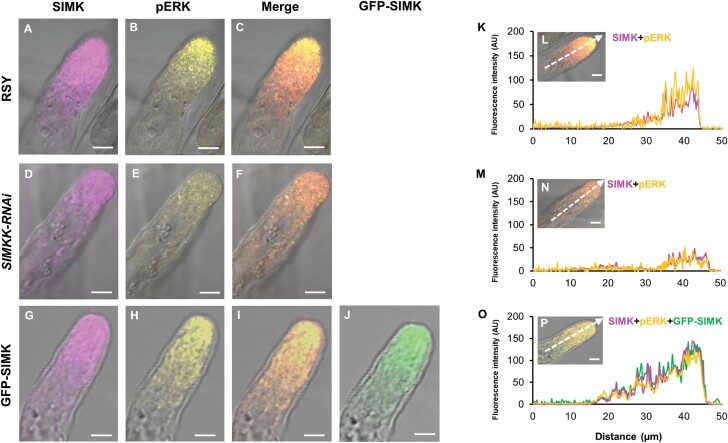
Subcellular immunolocalization of SIMK and activated MAPKs in growing root hairs of alfalfa control and transgenic plants under control conditions. (A–J) Whole-mount immunofluorescence localization of SIMK and activated MAPKs in root hairs of alfalfa RSY plants (A–C) and plants of transgenic *SIMKK-RNAi* (D–F) and GFP-SIMK (G–J) lines. SIMK (in magenta) was immunostained with SIMK-specific antibody (A, D, G) and activated MAPKs (pERK, in yellow) using phospho-specific pERK 44/42 antibody (B, E, H). Overlay of bright-field images with fluorescence channels (C, F, I). GFP-tagged SIMK (in green) was localized via GFP fluorescence in a fixed root hair of the transgenic GFP-SIMK line (J). (K, M, O) Fluorescence intensity profiles of SIMK, activated MAPKs, and GFP-tagged SIMK distribution along the measured line shown in (L, N, P). Scale bar=5 µm (A–J, L, N, P).

To characterize SIMK localization patterns in growing root hairs of alfalfa control and transgenic plants, whole-mount immunofluorescence analysis after plant fixation was performed. Under control conditions, when alfalfa root hairs were not exposed to *E. meliloti*, a tip-focused pattern of SIMK distribution was observed (Fig. 1). Immunodetection revealed mainly apical and subapical localization of SIMK in growing root hairs of alfalfa RSY plants (Fig. 1A) and plants of transgenic *SIMKK-RNAi* (Fig. 1D) and GFP-SIMK (Fig. 1G) lines. Moreover, the activated pools of MAPKs in root hairs of RSY (Fig. 1B), *SIMKK-RNAi* (Fig. 1E), and GFP-SIMK (Fig. 1H) plants were spatially detected, showing the same distribution (Fig. 1C, F, I). GFP localization in fixed root hairs of the transgenic GFP-SIMK line confirmed the SIMK localization pattern obtained by immunolabelling (Fig. 1G, J). Profiling of fluorescence intensity distribution along individual root hairs was documented by semi-quantitative measurements, showing higher accumulation of SIMK and activated MAPKs in the apex and subapex of alfalfa root hairs (Fig. 1K–P). In comparison with RSY root hairs (Fig. 1K, L), the displayed profile distribution revealed decreased fluorescence intensity of both SIMK and activated MAPKs in root hair tips of the transgenic *SIMKK-RNAi* line (Fig. 1M, N). In contrast, the fluorescence intensity of both SIMK and activated MAPKs was increased in root hair tips of the transgenic GFP-SIMK line overexpressing GFP-tagged SIMK (Fig. 1O, P). These results demonstrate a considerably decreased presence of activated SIMK in root hair tips of the transgenic *SIMKK-RNAi* line compared with RSY. At the same time, it was considerably increased in the overexpression GFP-SIMK line. Since root colonization by *E. meliloti* initiates from growing root hairs, the presence of activated SIMK in the root hair tip may be an essential component of initial attachment and invasion steps by rhizobia. It could be potentially required for the establishment and efficient formation of early symbiotic structures.

### The reaction of GFP–SIMK to *E. meliloti* infection

**Fig. 2.**
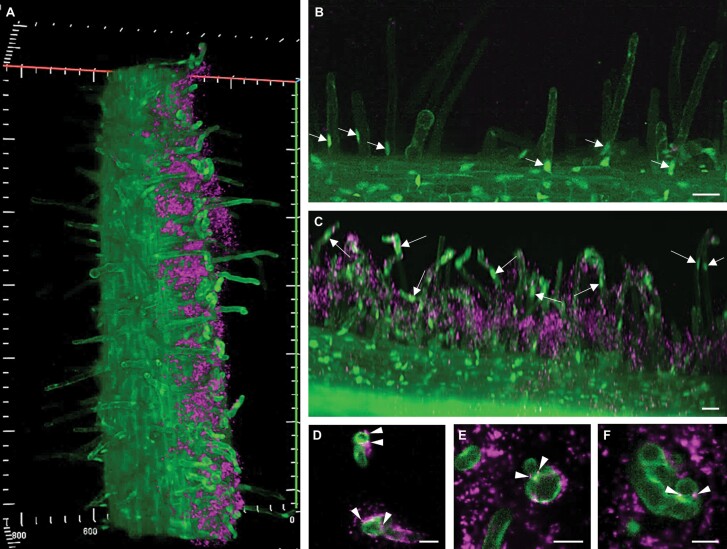
Live-cell imaging of early nodulation stages in roots of the transgenic GFP-SIMK line at 3 dpi with mRFP-labelled *E. meliloti* using LSFM. (A) 3D rendering overview of alfalfa root stably expressing GFP-tagged SIMK (green) co-cultivated with mRFP-labelled rhizobia (magenta). Rhizobia growing on the surface of agar plates are associated with a portion of the root and root hairs that were in contact with the agar plate surface. (B) Position of nuclei in root hairs not interacting with rhizobia (arrows). (C) Position of nuclei in root hairs interacting with rhizobia (arrows). (D–F) Details of root hair infection during rhizobia attachment and internalization (arrowheads). Scale bar=20 µm (D–F), 40 µm (B, C).

To characterize the GFP–SIMK localization pattern during early nodulation stages *in vitro*, live-cell imaging of alfalfa roots stably expressing GFP-tagged SIMK, co-cultivated with mRFP-labelled *E. meliloti*, was performed by LSFM at 3–4 dpi. The mode of interaction was analysed in the non-elongating part of roots, mainly involved in early root hair–*E. meliloti* interaction and nodulation according to microscopic examination (Supplementary Fig. S1C). In all plants included in the analysis, this zone was located more or less at the same distance from the root apex. In this root zone before *E. meliloti* application, GFP–SIMK was not accumulated in the tip of root hairs because of terminated tip growth (Fig. 2A, B). GFP–SIMK is at this stage located mainly in nuclei and cortical cytoplasm of largely vacuolated root hairs (Fig. 2B). Roots inoculated with *E. meliloti* were cultivated on the surface of agar plates, leading to the formation of a dense layer-like biofilm of mRFP-labelled *E. meliloti* associated only with the root and root hairs touching the agar surface. This model allows the simultaneous and independent study of root hairs symbiotically interacting with rhizobia, but also root hairs untouched by rhizobia that were exposed to air inside the Petri dish. Both types of root hairs are present on the same root exposed to the same conditions and treatments (Fig. 2A). Therefore, 3D rendering of symbiotically infected GFP-SIMK root enabled us to distinguish not only non-interacting alfalfa root hairs from those that interact with rhizobia but also the position of their nuclei with respect to infection (Supplementary Video S1). In non-growing root hairs that were not in physical contact with rhizobia, nuclei were positioned almost uniformly near the root hair base (Fig. 2B; Supplementary Video S1), while in reactivated root hairs under symbiotic interaction, nuclei were located closer to the site of rhizobia attachment at the tip (Fig. 2C; Supplementary Video S1). Root hairs not touching the agar surface and sticking out into the air having no contact with *E. meliloti* in treated roots might behave differently from root hairs touching a wet agar surface in bacteria-free conditions. To avoid this option, we analysed this on non-inoculated roots. In the root zone close to the root apex where root hairs touching the agar surface are still actively growing (normally not reacting to the presence of *E. meliloti*), nuclei in root hairs were located close to the root hair tip. In this case, the tip and subapical part of root hairs were filled with GFP–SIMK (Supplementary Fig. S2A–D). In the non-elongating portion of the root at a distance from the root apex where root hairs touching the agar surface terminated their growth (typically involved in reactions with *E. meliloti*), nuclei were located at the basal part and tips of root hairs were filled with vacuole, restricting GFP–SIMK to a thin cortical layer (Supplementary Fig. S2E–H). Therefore, we did not find a difference between root hairs touching a wet agar surface in non-inoculated roots, and root hairs sticking out into the air without contact with *E. meliloti* in treated roots. Preference for detailed LSFM live-cell imaging was given to the curled root hairs with attached rhizobia (Fig. 2D) or rhizobia already enclosed in the root hair curls (Fig. 2E, F), for characterization of SIMK involvement in early infection.

### *E. meliloti*-induced GFP–SIMK subcellular relocation

**Fig. 3.**
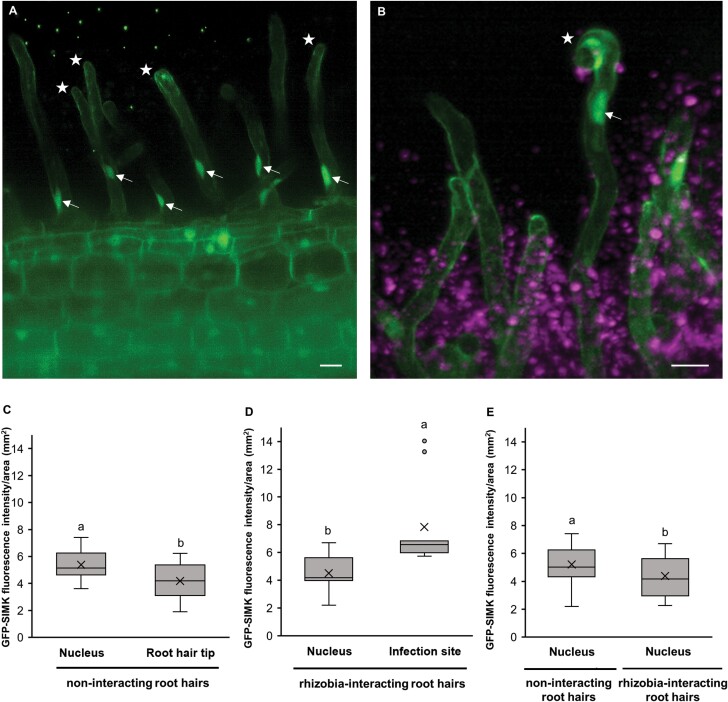
Quantitative analysis of GFP–SIMK fluorescence intensity distribution in nuclei and tips of alfalfa root hairs under control conditions and upon symbiotic interaction with mRFP-labelled *E. meliloti*. (A and B) GFP–SIMK distribution in non-interacting root hairs (A) and root hairs symbiotically interacting with rhizobia (B). Measurement of GFP–SIMK mean fluorescence intensity was performed in nuclei (arrows) and root hair tips (asterisks) of non-interacting root hairs (A) and in nuclei (arrows) and infection sites (asterisks) of interacting root hairs (B). (C–E) Quantitative evaluation of GFP-SIMK signal intensity in non-interacting root hairs (C, *n*=15) and in interacting root hairs (D, *n*=18), and comparison of GFP–SIMK signal intensity in nuclei of non-interacting and interacting root hairs (E, *n*=19). Box plots display the first and third quartiles, split by the median; the crosses indicate the mean values; whiskers extend to include the max/min values. Lowercase letters indicate statistical significance between lines according to one-way ANOVA with post-hoc Tukey HSD test (*P*<0.05). Green dots in (A) are fiducial markers. Scale bar=20 µm (A, B).

To find out whether exposure of alfalfa plants to beneficial rhizobia leads to changes in GFP–SIMK distribution that could be related to the early symbiotic interaction, the mean fluorescence intensity of GFP–SIMK was quantitatively evaluated in non-interacting (Fig. 3A) and *E. meliloti*-interacting (Fig. 3B) root hairs. Under control conditions, GFP–SIMK was present in nuclei and also in the cytoplasm within vacuolated root hair tips of non-interacting root hairs. Nevertheless, a higher amount of GFP–SIMK was detected in nuclei (Fig. 3C). During early nodulation stages, GFP–SIMK was localized in nuclei, but substantial accumulation also occurred around rhizobia at specific infection sites (Fig. 3B). Upon this early rhizobial infection, the amount of GFP–SIMK in nuclei decreased compared with control conditions (Fig. 3D, E). It seems that GFP–SIMK rather redistributes in root hairs upon rhizobia interaction and accumulates at infection sites where the nodulation process begins (Fig. 3B, D). This finding further suggests the supportive role of SIMK during the early stages of alfalfa nodulation.

### Association of GFP–SIMK with *E. meliloti* infection sites

**Fig. 4.**
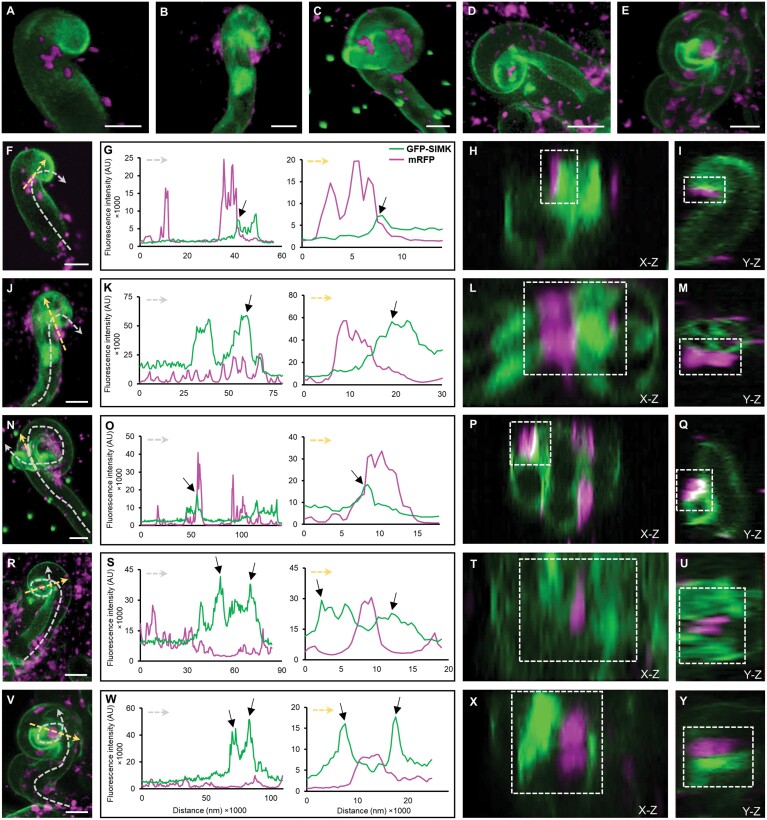
Live-cell localization of GFP–SIMK and its association with mRFP-labelled *E. meliloti* during early nodulation stages in root hairs 3–4 dpi using LSFM. (A–E) 3D rendering of selected root hairs at early sequential infection stages showing the distribution of GFP–SIMK (green) and rhizobia (magenta) during attachment to the root hairs (A), followed by rhizobia proliferation and early internalization (B, C), infection pocket formation (D), and rhizobia enclosure inside infection pockets (E). (F–Y) Detailed qualitative and semi-quantitative analysis of GFP–SIMK (green) and rhizobia (magenta) distribution during attachment to the root hairs (F–I), rhizobia proliferation and early internalization (J–Q), infection pocket formation (R–U), and rhizobia enclosure inside infection pockets (V–Y). *n*=3 root hairs per stage from three independent roots. Semi-quantitative evaluation of GFP–SIMK and mRFP-labelled rhizobia fluorescence distribution (G, K, O, S, W) along dashed arrows in (F, J, N, R,V ), indicating GFP–SIMK distribution in symbiotically infected root hairs (grey dashed arrows) and its association with rhizobia at specific infection sites (yellow dashed arrows). Representative images prepared from orthogonal projections in *X–Z* views (H, L, P, T, X) and *Y–Z* views (I, M, Q, U, Y) show a detailed view of GFP–SIMK accumulation around fluorescently labelled rhizobia (marked with white dashed boxes). Black arrows in (G, K, O, S, W) show increased accumulation of GFP–SIMK. Green dots in (C, N) are fiducial markers. Root hairs displayed in 3D rencering (A–E), single optical sections (F, J, N, R, V) and orthogonal projections in X–Z and Y–Z views Images A and F, B and J, C and N, D and R, and E and V show the same root hairs, labelled with dashed arrows for fluorescence profile measurements in later cases. Scale bar=10 µm (F, J, N, R, V).

Detailed live-cell imaging of symbiotically interacting root hairs revealed localization of GFP–SIMK and its association with the position of fluorescently labelled *E. meliloti* at individual early infection stages, beginning from rhizobia attachment (Fig. 4A, B), rhizobia entry into the root hairs (Fig. 4B), infection pocket formation (Fig. 4D), up to complete enclosure of rhizobia inside infection pockets (Fig. 4E). The first morphological response to attached rhizobia was root hair curling (Fig. 4A, F). Semi-quantitative evaluation of GFP–SIMK fluorescence intensity distribution showed increased accumulation of GFP–SIMK in the apex of curled root hairs, but also at a specific site of rhizobia attachment (Fig. 4F, G). Moreover, orthogonal projections revealed a very close association of GFP–SIMK with rhizobia attached to the root hair at this specific infection site in the *X*–*Z* view (Fig. 4H; Supplementary Video S2 at 0:00:14 s–0:00:21 s) and the *Y*–*Z* view (Fig. 4I; Supplementary Video S3 at 0:00:14 s−0:00:21 s). Later upon infection, a cluster of rhizobia was located specifically at the neck of the root hair curl where rhizobia will enter the root hair (Fig. 4J). Profile measurements revealed an accumulation of GFP–SIMK in the nucleus located close to the infection site, its relocation from the apex, and specific accumulation at the infection site (Fig. 4J, K). Orthogonal projections from the *X–Z* view (Fig. 4L; Supplementary Video S4 at 0:00:14 s−0:00:18 s) and the *Y–Z* view (Fig. 4M; Supplementary Video S5 at 0:00:16 s−0:00:24 s) revealed close association of GFP–SIMK with rhizobia gathered at the root hair curl through which rhizobia internalization typically takes place. Before rhizobia entry into the alfalfa root hairs, a stage of very tight contact between the curled root hair tip and entrapped rhizobia was captured by LSFM (Fig. 4N). Profile measurements showed increased fluorescence intensity of GFP–SIMK at the site where rhizobia are in very close contact with the root hair (Fig. 4N, O). Observation of very tight association including the partial overlay indicating an internalization was confirmed from orthogonal projections in the *X–Z* view (Fig. 4P; Supplementary Video S6 at 0:0014 s−0:00:21 s) and the *Y–Z* view (Fig. 4Q, Supplementary Video S7 at 0:00:15 s−0:00:24 s). Once individual rhizobia were entrapped inside a root hair curl, formation of an infection pocket was initiated (Fig. 4R). GFP–SIMK was found to be accumulated around rhizobia surrounding them inside root hair curls (Fig. 4S) and associated with them as shown from orthogonal projections in the *X–Z* view (Fig. 4T; Supplementary Video S8 at 0:00:13 s−0:00:25 s) and the *Y–Z* view (Fig. 4U; Supplementary Video S9 at 0:00:14 s−0:00:22 s). Later, rhizobia divide and form colonies within infection pockets (Fig. 4V) from which ITs are subsequently initiated. GFP–SIMK was strongly accumulated around infection pockets containing rhizobia (Fig. 4W), and orthogonal projections in the *X–Z* view (Fig. 4X; Supplementary Video S10 at 0:00:15 s−0:00:22 s) and the *Y–Z* view (Fig. 4Y; Supplementary Video S11 at 0:00:14 s−0:00:21 s) corroborated close GFP–SIMK distribution around infection pockets. The position of lines, along which the semi-quantitative evaluation of fluorescence distribution has been analysed, was decided according to 3D rendering (Fig. 4A–E), images prepared from orthogonal projections in *X–Z* views (Fig. 4H, L, P, T, X) and *Y–Z* views (Fig. 4G, K, O, S, W), and 3D visualization of the whole acquired volume with dynamic orthogonal sectioning from an *X–Z* view (Supplementary Videos S2, S4, S6, S8, S10) and from a *Y–Z* view (Supplementary Videos S3, S5, S7, S9, S11). Images prepared from orthogonal projections in *X–Z* views (Fig. 4H, L, P, T, X) and *Y–Z* views (Fig. 4G, K, O, S, W) locate attached or internalized rhizobia quite precisely, and profiles presented in Fig. 4G, K, O, S, and W correspond well with this localization pattern. Importantly, *in vivo* time-lapse imaging showed that accumulation of GFP–SIMK at the infection site in root hairs during rhizobia attachment (Supplementary Video S12) and around infection pockets (Supplementary Video S13) is stable over time, as semi-quantitatively documented profile measurements of GFP–SIMK fluorescence intensity distribution did not fluctuate (Supplementary Videos S14, S15). Altogether, live-cell LSFM imaging showed specific localization and accumulation of GFP–SIMK at infection sites during the early infection stages, which very closely associated with attaching and internalizing rhizobia. Based on the presence of activated SIMK in the growing tips of alfalfa root hairs (Fig. 1), it seems that accumulation and activation of SIMK might play an important role in the early stages of alfalfa root hair infection by *E. meliloti*.

### SIMK subcellular localization during formation of infection pockets

**Fig. 5.**
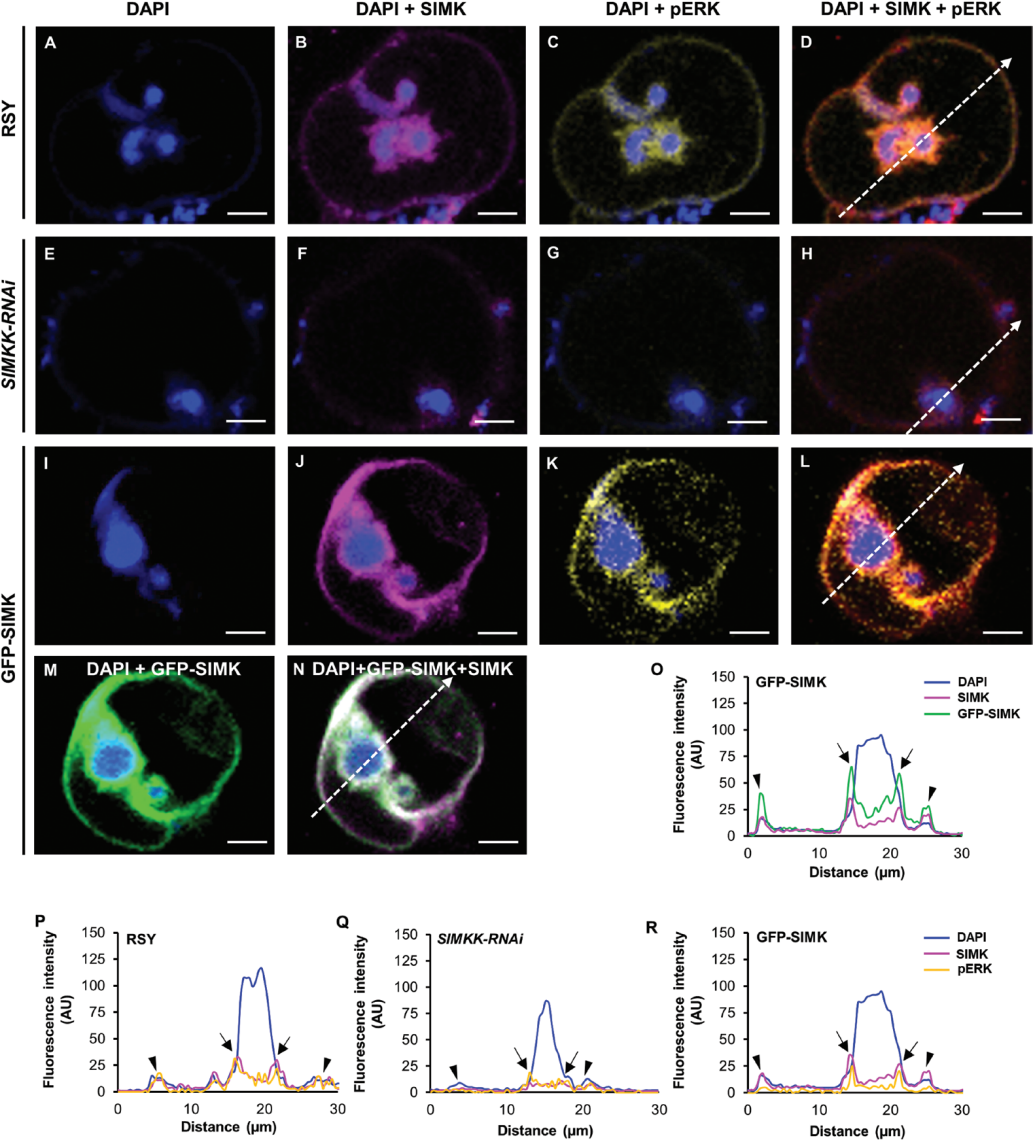
Subcellular immunolocalization of SIMK and activated MAPKs around infection pockets in curled root hairs after inoculation with *E. meliloti*. (A, E, I) Localization of DAPI-stained rhizobia inside an infection pocket of RSY (A), *SIMKK-RNAi* (E), and GFP-SIMK (I) lines. (B, F, J) SIMK immunostained with SIMK-specific antibody and overlaid with DAPI in RSY (B), *SIMKK-RNAi* (F), and GFP-SIMK (J) lines. (C, G, K) Activated MAPKs immunostained with phospho-specific pERK 44/42 antibody and overlaid with DAPI in RSY (C), *SIMKK-RNAi* (G), and GFP-SIMK (K) lines. (D, H, L) Overlay of DAPI, SIMK, and activated MAPKs in RSY (D), *SIMKK-RNAi* (H), and GFP-SIMK (L) lines. (M, N) GFP-tagged SIMK overlaid with DAPI (M), and overlay of GFP-tagged SIMK, SIMK immunostained with SIMK-specific antibody, and DAPI in the transgenic GFP-SIMK line (N). (O–R) The fluorescence intensity distribution of SIMK, activated MAPKs, GFP-tagged SIMK, and DAPI was measured along profiles indicated by white dashed arrows in (D, H, L, N). Black arrows indicate the membrane around the infection pocket; black arrowheads indicate the root hair plasma membrane. Scale bar=5 µm (A–N).

To reveal the subcellular localization pattern of SIMK and activated MAPKs in root hairs of control RSY and transgenic *SIMKK-RNAi* and GFP-SIMK plants 3–7 dpi with *E. meliloti*, and their association with infection pockets, immunofluorescence localization microscopy was employed. Fixed root samples were immunolabelled for SIMK and activated MAPKs using SIMK-specific and phospho-specific antibodies, respectively. The pattern of SIMK and activated MAPKs localization was documented in infection pockets, the first symbiotic structure, formed inside root hairs after rhizobial internalization. DAPI, typically used for DNA nuclear staining, stained effectively also *E. meliloti*, which enabled a detailed study of MAPKs association with intracellular compartments enclosing rhizobia during the early stages of the nodulation process. Upon root hair curling, *E. meliloti* was entrapped in alfalfa root hair curls and became completely enclosed inside infection pockets (Fig. 5A). At this symbiotic stage, immunostaining revealed SIMK localization close to the plasma membrane and particularly prominent SIMK-specific accumulation around infection pockets in the alfalfa RSY line (Fig. 5B). Labelling of activated MAPKs showed the same pattern of localization (Fig. 5C), indicating a co-localization with SIMK-specific signal (Fig. 5D). This suggests that MAPKs localized around infection pockets were phosphorylated. Moreover, a semi-quantitative evaluation of fluorescence intensity distribution confirmed the close association of both SIMK and activated MAPKs with infection pockets (Fig. 5P).

In root hairs of the transgenic *SIMKK-RNAi* line, infection pockets filled with DAPI-stained *E. meliloti* (Fig. 5E) were surrounded by a very faint signal of both SIMK (Fig. 5F) and activated MAPKs (Fig. 5G), showing a similar pattern of localization (Fig. 5H). Semi-quantitative profile measurements revealed an association of SIMK and activated MAPKs with infection pockets. However, compared with the alfalfa RSY plants (Fig. 5P), the fluorescence intensity of SIMK and activated MAPKs was substantially decreased in the transgenic *SIMKK-RNAi* line (Fig. 5Q).

Similarly, inside root hairs of the transgenic GFP-SIMK line, when *E. meliloti* became fully entrapped inside infection pockets (Fig. 5I), immunodetection with SIMK-specific antibody revealed substantial accumulation mainly around infection pockets, but prominent SIMK-specific signal was also detected at the plasma membrane of curled root hairs (Fig. 5J). Activated MAPKs showed a similar subcellular localization pattern around infection pockets and at the plasma membrane of curled root hairs (Fig. 5K), leading to a high degree of co-localization with SIMK-specific signal (Fig. 5L). The SIMK localization pattern obtained by immunostaining with SIMK-specific antibody was independently confirmed by localization of GFP-tagged SIMK in curled root hairs of the GFP-SIMK line (Fig. 5M). Also, GFP-tagged SIMK showed a high degree of co-localization with SIMK signal obtained by immunolabelling with SIMK-specific antibody (Fig. 5N), and semi-quantitative evaluation of SIMK and activated MAPKs fluorescence intensity distribution clearly revealed its close association with infection pockets (Fig. 5O, R).

**Fig. 6.**
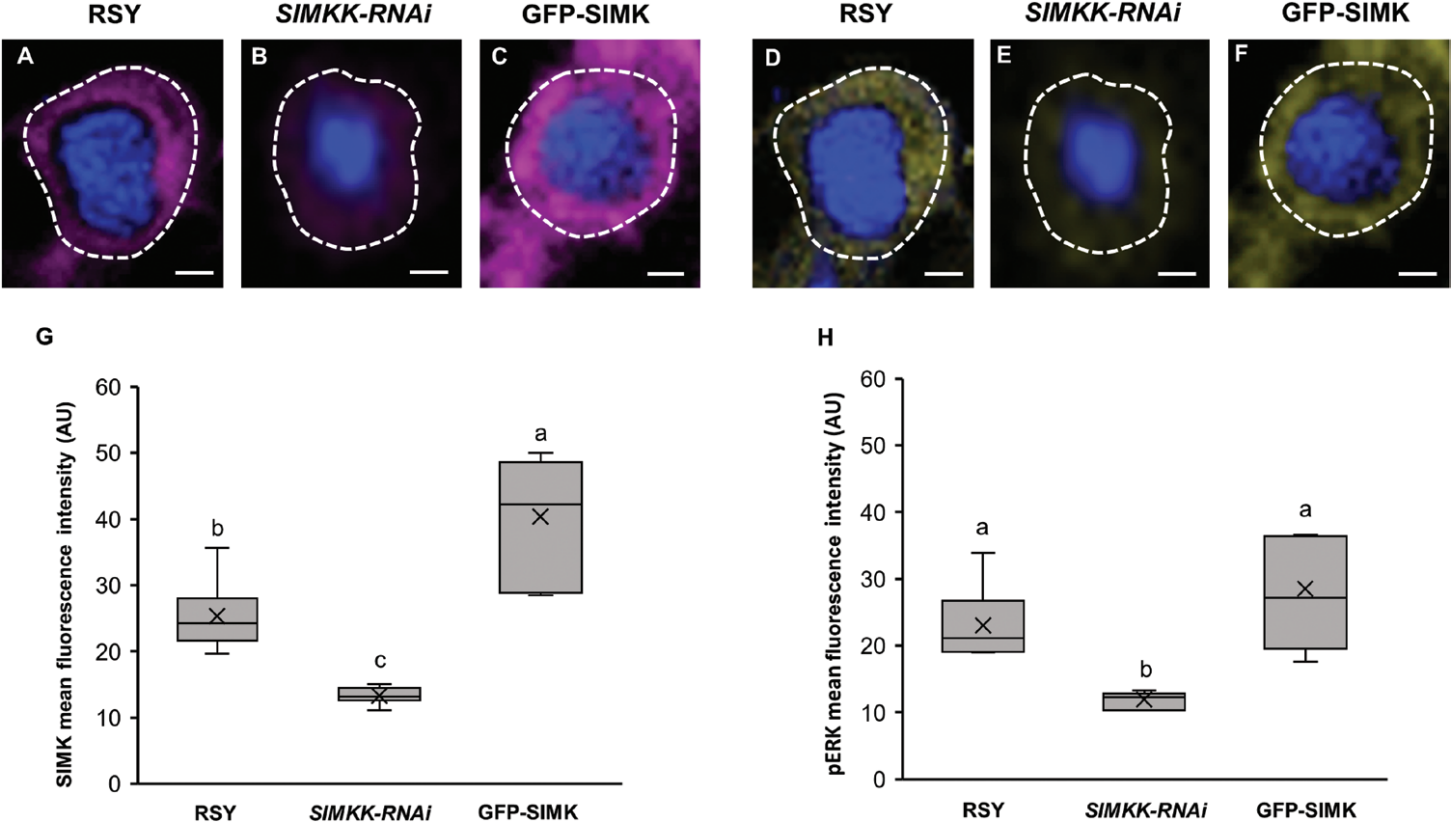
Quantitative analysis of the fluorescence intensity distribution of SIMK and phosphorylated MAPKs around infection pockets in curled root hairs after inoculation with *E. meliloti*. (A–F) Immunolocalization of SIMK (A–C) and phosphorylated MAPKs (D–F) in infection pockets of RSY (A, D; *n*=10 for SIMK, *n*=7 for pERK), *SIMKK-RNAi* (B, E; *n*=10 for SIMK, *n*=7 for pERK), and GFP–SIMK (C, F; *n*=9 for SIMK, *n*=7 for pERK) lines. White dashed lines in (A–F) indicate defined ROIs in which the mean fluorescence intensity was measured. (G, H) Quantitative evaluation of the signal intensity of SIMK (G) and activated MAPKs (H) in transgenic *SIMKK-RNAi* and GFP-SIMK lines compared with RSY plants. Box plots display the first and third quartiles, split by the median; the crosses indicate the mean values; whiskers extend to include the max/min values. Lowercase letters indicate statistical significance between lines according to one-way ANOVA with post-hoc Tukey HSD test (*P*<0.05). Scale bar=2 µm (A–F).

In addition, quantitative comparative analysis of mean fluorescence intensity around infection pockets revealed, in comparison with control RSY (Fig. 6A, G), significantly higher levels of SIMK in GFP-SIMK plants (Fig. 6C, G) and significantly lower levels in the transgenic *SIMKK-RNAi* line (Fig. 6B, G). Also, a lower level of activated MAPKs was found around infection pockets inside root hairs of the transgenic *SIMKK-RNAi* line (Fig. 6E, H), while no significant difference was observed in the transgenic GFP-SIMK line compared with alfalfa RSY plants (Fig. 6D, F, H).

Quantitative determination of the co-localization rate between signals of SIMK and activated MAPKs, expressed by Pearson’s correlation coefficient, revealed the highest value around infection pockets in the transgenic GFP-SIMK line and the lowest in the transgenic *SIMKK-RNAi* line (Supplementary Fig. S3).

This analysis clearly showed SIMK-specific accumulation and activation around infection pockets containing entrapped *E. meliloti* in alfalfa root hairs. The level of active SIMK accumulation was strongly associated with the SIMK expression level. It was substantial in root hairs of the transgenic GFP-SIMK line, while the lowest presence of active SIMK was detected around infection pockets in the transgenic *SIMKK-RNAi* line, causing strong down-regulation of both *SIMKK* and *SIMK* (Hrbáčková *et al.*, 2021). Since infection pockets represent the site of *E. meliloti* entry and IT initiation, these results indicate that active SIMK accumulated at this specific location might be required for efficient IT formation.

### SIMK subcellular localization during formation of ITs

**Fig. 7.**
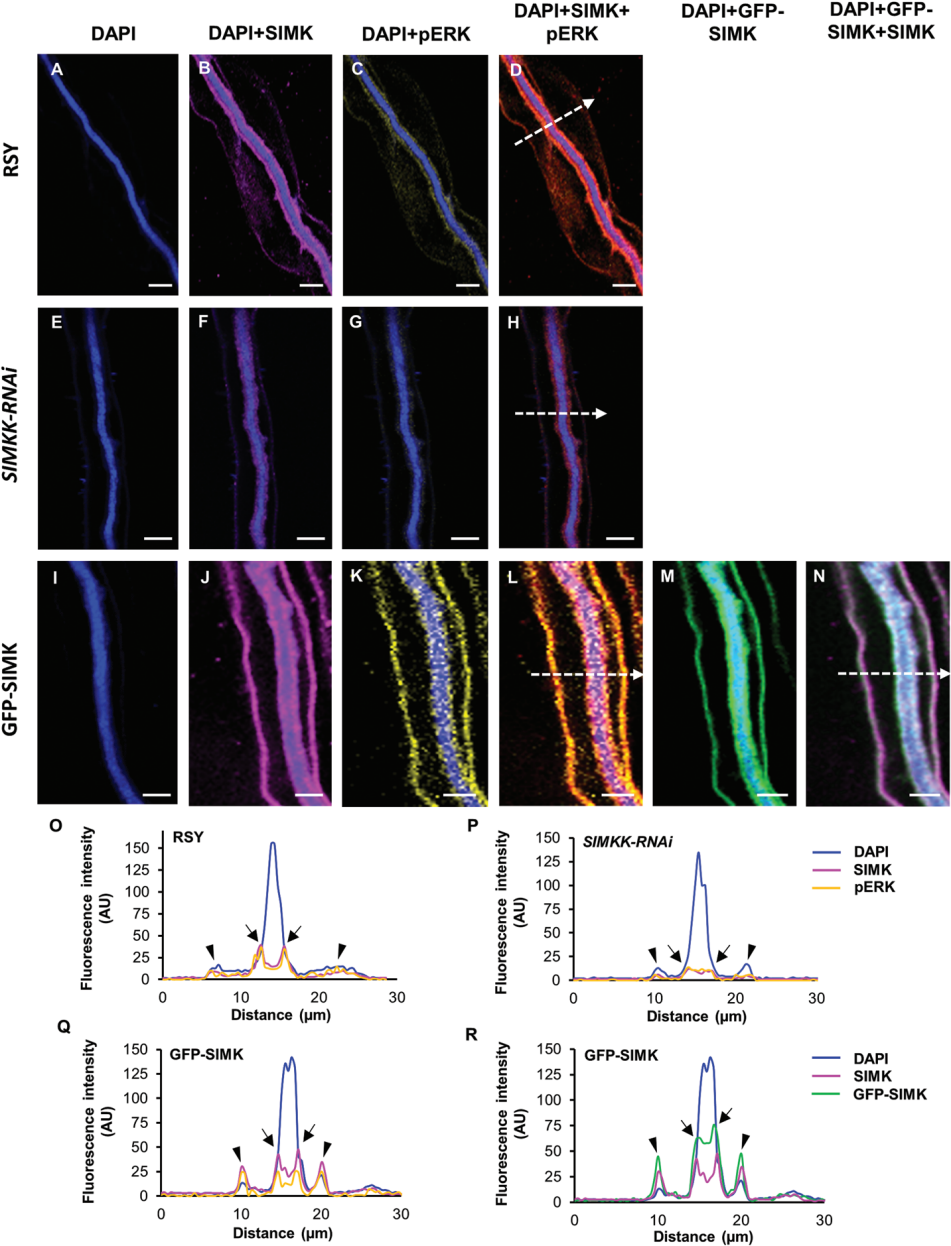
Subcellular immunolocalization of SIMK and activated MAPKs around ITs in root hairs induced after inoculation with *E. meliloti*. (A, E, I) Localization of DAPI-stained rhizobia inside ITs of RSY (A), *SIMKK-RNAi* (E), and GFP-SIMK (I) lines. (B, F, J) SIMK immunostained with SIMK-specific antibody and overlaid with DAPI in RSY (B), *SIMKK-RNAi* (F), and GFP-SIMK (J) lines. (C, G, K) Activated MAPKs immunostained with phospho-specific pERK 44/42 antibody and overlaid with DAPI in RSY (C), *SIMKK-RNAi* (G), and GFP-SIMK (K) lines. (D, H, L) Overlay of DAPI, SIMK, and activated MAPKs in RSY (D), *SIMKK-RNAi* (H), and GFP-SIMK (L) plants. (M, N) GFP-tagged SIMK overlaid with DAPI (M) and overlay of GFP-tagged SIMK, SIMK immunostained with SIMK-specific antibody, and DAPI in the transgenic GFP-SIMK line (N). (O–R) The fluorescence intensity distribution of SIMK, activated MAPKs, GFP-tagged SIMK, and DAPI was measured along profiles indicated by white dashed arrows in (D, H, L, N). Black arrows indicate the membrane around ITs, black arrowheads indicate the plasma membrane of root hairs. Scale bar=5 µm (A–N).

Complete enclosure of rhizobia inside infection pockets is followed by an invagination of the host cell plasma ­membrane and initiation of tunnel-like ITs. Therefore, the subcellular localization pattern of SIMK and activated MAPKs was characterized by immunolabelling during IT formation and propagation through root hairs. Inside root hairs of the alfalfa RSY line, ITs were easily detectable owing to DAPI-stained *E. meliloti* (Fig. 7A). Immunostaining revealed SIMK-specific signals surrounding growing ITs (Fig. 7B). Activated MAPKs immunolabelled with anti-phospho-p44/42 antibody showed the same subcellular distribution (Fig. 7C), leading to a high degree of co-localization with the SIMK signal (Fig. 7D). This co-localization pattern suggests that SIMK located around ITs was phosphorylated. Semi-quantitative analysis of the fluorescence intensity distribution revealed a close association of both SIMK and activated MAPKs with the surface of ITs (Fig. 7O).

In the transgenic *SIMKK-RNAi* line, ITs filled with *E. meliloti* (Fig. 7E) were similarly decorated by MAPKs, but showed a very weak signal of both SIMK (Fig. 7F) and activated MAPKs (Fig. 7G). Nevertheless, the distribution pattern indicated their subcellular co-localization (Fig. 7H). Profiling of the fluorescence intensity distribution of SIMK and activated MAPKs revealed their association with ITs, but substantially decreased (Fig. 7P).

In the case of ITs in the transgenic GFP-SIMK line (Fig. 7I), immunostaining with SIMK-specific antibody revealed a strong accumulation of SIMK not only along ITs, but also at the plasma membrane of root hairs (Fig. 7J). Signal specific for activated MAPKs showed the same subcellular localization pattern (Fig. 7K) and co-localized with SIMK signal (Fig. 7L). Observation of GFP-tagged SIMK along ITs (Fig. 7M) confirmed the localization pattern obtained by immunolabelling with SIMK-specific antibody and co-localized with SIMK signal (Fig. 7N). Semi-quantitative evaluation of fluorescence intensity along the indicated profiles (Fig. 7L, N) confirmed enhanced and close association of SIMK and activated MAPKs with ITs and plasma membrane of root hairs (Fig. 7Q, R).

**Fig. 8.**
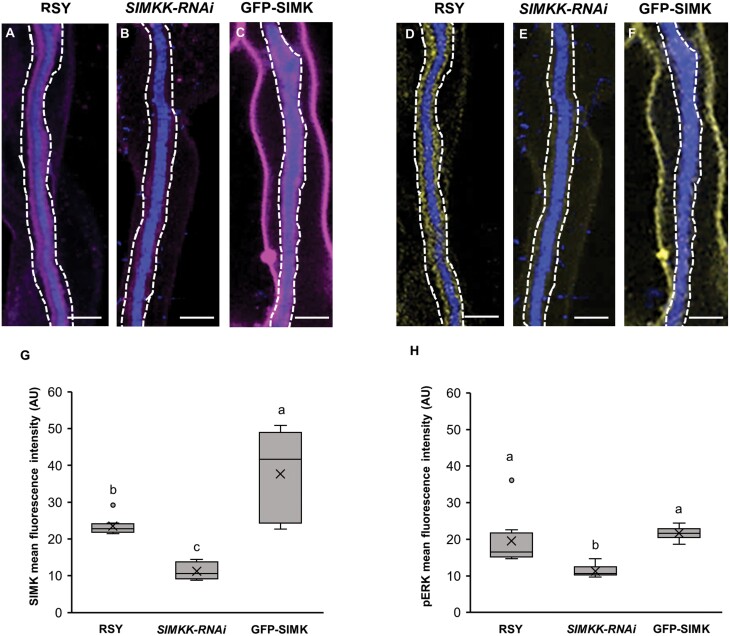
Quantitative analysis of the fluorescence intensity distribution of SIMK and phosphorylated MAPKs around ITs in root hairs after inoculation with *E. meliloti*. (A–F) Immunolocalization of SIMK (A–C) and phosphorylated MAPKs (D–F) in ITs of RSY (A, D; *n*=8 for SIMK, *n*=8 for pERK), *SIMKK-RNAi* (B, E; *n*=6 for SIMK, *n*=7 for pERK), and GFP-SIMK (C, F; *n*=8 for SIMK, *n*=8 for pERK) lines. White dashed lines in (A–F) indicate defined ROIs in which the mean fluorescence intensity was measured. (G, H) Quantitative evaluation of the signal intensity of SIMK (G) and activated MAPKs (H) in transgenic *SIMKK-RNAi* and GFP-SIMK lines compared with RSY plants. Box plots display the first and third quartiles, split by the median; the crosses indicate the mean values; whiskers extend to include the max/min values. Lowercase letters indicate statistical significance between lines according to one-way ANOVA with post-hoc Tukey HSD test (*P*<0.05). Scale bar=5 µm (A–F).

Moreover, the amount of SIMK (Fig. 8A–C) and activated MAPKs (Fig. 8D–F) determined by quantitative analysis of mean fluorescence intensity around ITs was markedly lower in plants of the transgenic *SIMKK-RNAi* line in comparison with alfalfa RSY and GFP-SIMK plants (Fig. 8G, H). In contrast, the amount of activated MAPKs in the transgenic GFP-SIMK line was similar to that of RSY plants (Fig. 8H).

The co-localization rate between SIMK and activated MAPKs was quantitatively determined by Pearson’s correlation coefficient, revealing that the overall co-localization rate between SIMK and activated MAPKs was significantly higher along ITs in the transgenic GFP-SIMK line and RSY plants compared with the transgenic *SIMKK-RNAi* plants (Supplementary Fig. S4). In control, uninfected root hairs with terminated tip growth, subcellular immunolocalization of SIMK with SIMK-specific antibody and activated MAPKs with phospho-specific pERK 44/42 antibody revealed their co-localization. However, no particular subcellular accumulation of SIMK, activated MAPKs, or GFP–SIMK in root hairs of RSY, *SIMKK-RNAi*, and GFP-SIMK lines have been observed (Supplementary Fig. S5).

Immunolocalization together with semi-quantitative and quantitative co-localization analyses clearly revealed the presence of SIMK-specific signal along ITs in alfalfa root hairs. Increased accumulation of active SIMK along ITs was observed in the transgenic GFP-SIMK line, while the lowest accumulation was detected in the *SIMKK-RNAi* line. All these data indicate that active SIMK might be involved during IT formation and its growth towards the site of root nodule primordia initiation. Therefore, the supportive role of SIMK in the propagation of rhizobia-filled ITs through plant root hairs and cortex tissues might be important in the regulation and effectiveness of rhizobia delivery to the nodule primordium and subsequent nodule formation.

**Fig. 9.**
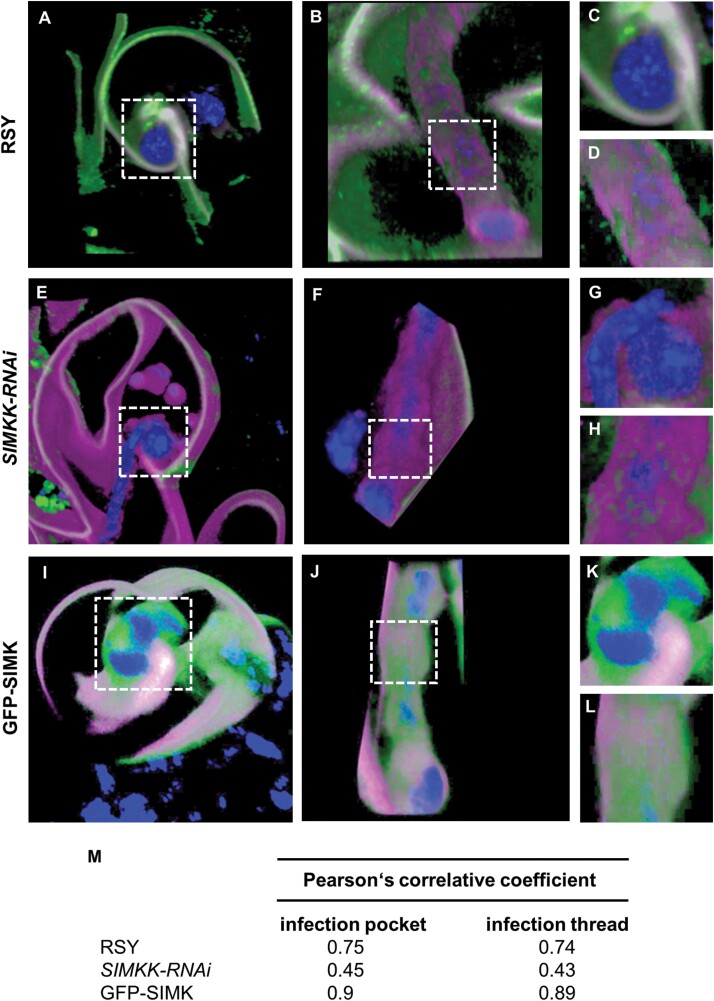
Volume 3D rendering of rhizobia-containing early symbiotic structures with immunolabelled SIMK and membranes visualized using FM4-64FX in root hairs after inoculation with *E. meliloti*. (A–D) RSY root hairs. (E–H) Root hairs of the *SIMKK-RNAi* line. (I–L) Root hairs of the GFP-SIMK line. Subcellular localization of SIMK with membranes of infection pockets (A, C, E, G, I, K) and ITs (B, D, F, H, J, L). Overlay of membranes (in magenta), SIMK (in green), and DAPI-stained rhizobia (in blue). (M) Averaged Pearson’s correlative coefficients of co-localization analysis between SIMK and FM4-64FX-stained membranes around infection pockets and ITs. Details of infection pockets and ITs shown in (C, D, G, H, K, L) are marked with a white dashed boxes in (A, B, E, F, I, J).

Moreover, we performed a visualization of plasma membranes using a fixable FM4-64FX, allowing precise observation of SIMK subcellular localization with regard to membranes of early symbiotic structures. Whole-mount immunofluorescence co-labelling in RSY revealed the presence of SIMK close to the membranous surface of infection pockets (Fig. 9A, C) and ITs (Fig. 9B, D). A lower amount of SIMK was found on membranes of infection pockets (Fig. 9E, G) and ITs (Fig. 9F, H) in the *SIMKK-RNAi* line, while a substantially increased amount of SIMK was accumulated on the surface of infection pockets (Fig. 9I, K) and ITs (Fig. 9J, L) in the GFP-SIMK line. In quantitative terms, Pearson’s correlation coefficient showed the highest co-localization rate between SIMK and FM4-64FX-labelled membranes in the GFP-SIMK line. In contrast, the degree of co-localization was considerably decreased in the *SIMKK-RNAi* line (Fig. 9M). The data suggest a close association and interaction of SIMK with membranes of infection pockets and ITs during early nodulation stages.

### Involvement of active SIMK in formation of ITs

To correlate the presence of active SIMK around infection pockets and ITs with nodule formation, the efficiency of root hair infection by *E. meliloti* was examined. The number of ITs per the whole root system length was determined in alfalfa RSY (Supplementary Fig. S6A) and transgenic *SIMKK-RNAi* (Supplementary Fig. S6B) and GFP-SIMK (Supplementary Fig. S6C) plants at 10 dpi with *E. meliloti*, when actively growing ITs should already reach the root cortex. No significant difference was observed in the averaged root system length among the three respective lines at 10 dpi with *E. meliloti* (Supplementary Fig. S7A). However, the transgenic *SIMKK-RNAi* line, with strongly down-regulated *SIMKK* and *SIMK* (Hrbáčková *et al.*, 2021) and showing the lowest amount of active SIMK around infection pockets (Figs 6E, H, 9E,G,M), formed significantly fewer ITs compared with RSY and transgenic GFP-SIMK plants (Supplementary Fig. S7B), while the transgenic GFP-SIMK line produced a similar number of ITs to RSY plants (Supplementary Fig. S7B).

## Discussion

Leguminous plants are immensely important to the ecosystem and sustainable agriculture worldwide. Part of their success lies in their mutualistic partnership with beneficial nitrogen-­fixing bacteria, which helps them to manage nitrogen shortage and facilitate nutrient uptake (Brundrett, 2002; Bisseling and Geurts, 2020). In plants, developmental and cellular processes are regulated by MAPK-mediated phosphorylation cascades, and the activity of various protein kinases was shown to be also involved in symbiotic interactions and nodule formation (Grimsrud *et al.*, 2010; Komis *et al.*, 2018; Roy *et al.*, 2020). Although symbiotic nitrogen fixation is extensively studied in model legume species, such as *Medicago truncatula* and *Lotus japonicus*, little is known about the regulation of symbiotic interaction and the possible involvement of MAPK signalling in alfalfa nodulation. Here, using advanced LSFM, immunolocalization coupled with CLSM, and genetic engineering, we characterized the subcellular localization and activation pattern of SIMK, functionally modulating early stages of alfalfa interaction with *E. meliloti*.

Despite the remarkable progress in understanding MAPK regulation in plant development and immunity, the involvement and molecular mechanism of MAPK signalling cascades in various stages of symbiotic nodule development remain poorly understood and require in-depth investigation. In *L. japonicus*, a MAPKK, SIP2, was found to interact with a symbiosis receptor-like kinase (SymRK) and to play an essential role in early symbiotic signalling, nodule organogenesis, and development. Reduced expression of *SIP2* by RNAi impaired IT and nodule formation, resulting in reduced numbers of ITs, nodule primordia, and nodules (Chen *et al*., 2012). Yin *et al.* (2019) identified LjMPK6 as the phosphorylation target of SIP2 and showed that the SymRK–SIP2–LjMPK6 signalling module is required for nodule organogenesis and formation in *L. japonicus*. RNAi silencing of *LjMPK6* caused a lower ability of stable transgenic plants to form nodule primordia and nodules, while its overexpression promoted initiation of ITs, nodule primordia, and nodule formation. Histochemical β-glucuronidase (GUS) staining revealed promoter activity of *LjMPK6* in root hairs and nodule primordia early upon rhizobial infection but also during late symbiotic stages in all cells of young nodules and in the parenchyma and vascular bundles of mature nodules. In addition, a recent study demonstrated that LjPP2C, a type 2C protein phosphatase, fine-tunes nodule development in *L. japonicus* via dephosphorylating LjMPK6 (Yan *et al*., 2020). In *M. truncatula*, *MtMAPKK4* shows a high sequence identity to *MsSIMKK* and *LjSIP2*. Phenotypic characterization and nodulation assay with the heterozygous *mapkk4/+* knockout mutant showed that MtMAPKK4 is involved in plant growth, development, and nodule formation. Downstream interacting partners of MtMAPKK4 are MtMAPK3 and MtMAPK6 (Chen *et al*., 2017). Another MAPKK from *M. truncatula*, MtMAPKK5, directly activates MtMAPK3 and MtMAPK6 by phosphorylating the TEY motif within the activation loop. The stress signalling-mediated MtMAPKK5–MtMAPK3/6 module negatively affects the early symbiotic interaction of *M. truncatula* with beneficial soil bacteria, leading to the suppression of nodule formation (Ryu *et al.,* 2017). Interestingly, SIMKK shares 88% amino acid similarity with LjSIP2 (Chen *et al*., 2012) and also shows a high sequence identity to MtMAPKK4 (Chen *et al*., 2017). A better understanding of the involvement of MAPK cascades in the regulation of plant developmental and symbiotic processes in agronomically important legume crops still remains of great interest. In alfalfa, the role of the MsSIMKK–SIMK module is implicated in abiotic salt stress (Kiegerl *et al*., 2000; Ovečka *et al*., 2014) and in nodule formation, respectively (Hrbáčková *et al.*, 2021). SIMK overexpression leads to the development of longer root hairs and promoted ITs and nodule clustering (Hrbáčková *et al.*, 2021). In contrast, SIMK down-regulation was accompanied by the formation of shorter root hairs and few ITs and nodules. Moreover, SIMK overexpression promoted shoot biomass production and leaf and petiole development (Hrbáčková *et al.*, 2021). However, understanding of the SIMK subcellular localization and activation pattern clarifying the spatial and temporal model of SIMK involvement in alfalfa early nodulation stages remained unclear.

Possible SIMK involvement in alfalfa nodulation can be anticipated from its subcellular localization and activation during early symbiotic stages. Crucial input of the present study is the employment of a near-environmental advanced LSFM-based imaging method allowing gentle and non-invasive recordings of spatiotemporal interaction of intact root hairs with symbiotic rhizobia. Importantly, this innovative approach revealed a very tight association of SIMK with rhizobia docking, internalization, and symbiotic accumulation in infection pockets and ITs. We also confirmed that SIMK was activated in these locations. Previously, we have established LSFM for the spatiotemporal imaging of plant development at subcellular, cellular, tissue, and organ levels under controlled environmental conditions (Ovečka *et al*., 2018). In principle, the LSFM orthogonally positioned detection pathway with respect to the excitation pathway effectively eliminates out-of-focus fluorophore excitation, leading to the high signal-to-noise ratio of images and a spherical aberration-free detection. The light sheet utilized for the excitation is thin enough and allows fast optical sectioning of the whole sample volume. Therefore, high-speed acquisition of a large field of view allows observation of fast cellular processes in living organisms with high temporal resolution but low excitation energy. Voluminous samples might be challenging for LSFM, considering the depth of imaging, together with irregularities of light-sheet illumination induced by the absorption and diffraction of the light at the sample. However, these drawbacks can be alleviated by multiangle imaging. Thus, LSFM is an excellent method to study root hair development and offers the possibility to apply rhizobia to the imaging chamber by controlled perfusion. This innovative approach opens up new opportunities for studying plant–microbe interactions in real time and for the long term (Ovečka *et al*., 2022), which is indispensable for understanding regulation of the complex nodulation mechanism. LSFM was utilized in alfalfa to characterize root development (Vyplelová *et al*., 2018) and the involvement of the actin cytoskeleton in the interaction with *E. meliloti* (Ovečka *et al*., 2022). Live-cell imaging using LSFM clearly showed relocation of SIMK from root hair tips to the *E. meliloti* docking site and further close association with sites of rhizobia internalization. We also developed reliable immunolocalization protocols for whole-mount immunolabelling of root samples of *M. sativa*, achieving high signal efficiency and superb sample stability (Tichá *et al.*, 2020). Employing these immunolabelling methods explicitly adapted for alfalfa plantlets originating from somatic embryos, we were able to show, in addition to LSFM live-cell subcellular localization patterns of SIMK, also the localization of the activated MAPKs pool during the early stages of the nodulation process in alfalfa. Moreover, co-localization analysis of SIMK and phosphorylated MAPKs enabled us to check whether or not SIMK involved in alfalfa–*E. meliloti* early interaction is activated at the subcellular level. Previously, Šamaj *et al*. (2002) showed a tip-focused pattern of SIMK localization and activation in growing root hairs of alfalfa control plants. In the overexpression GFP-SIMK line, live-cell Airyscan CLSM imaging revealed subcellular localization of GFP–SIMK in root hair tips, but its activation state remained unknown (Hrbáčková *et al.*, 2021). In addition, biochemical methods showed an overall decreased accumulation of phosphorylated SIMK in the *SIMKK-RNAi* line; however, the detailed microscopic analysis focused on root hairs and SIMK localization and activation was not performed in the study (Bekešová *et al.*, 2015). Here, immunolocalization combined with quantitative analyses showed a tip-focused pattern of activated SIMK localization in growing root hairs of alfalfa RSY plants and plants of transgenic *SIMKK-RNAi* and GFP-SIMK lines. Decreased accumulation of activated SIMK in growing root hair tips was observed in the transgenic *SIMKK-RNAi* line. Although rhizobia can use different routes to invade plant roots, entrance via root hairs is probably the best understood and can be found in legumes such as alfalfa, soybean, pea, bean, and vetch (Sprent and James, 2007; Sprent, 2008; Ibáñez *et al.*, 2017). Since root hairs make the first contact with symbiotic rhizobia, active SIMK in root hairs may play an important role in alfalfa’s early interaction with *E. meliloti* during and after rhizobia attachment. We can only speculate on SIMK function during the early stages of interaction with *E. meliloti*. SIMK may be involved in the modulation and reorganization of the cytoskeleton upon attachment of bacteria, as the actin cytoskeleton is linked with SIMK in control of growing root hairs (Šamaj *et al.*, 2002), thus providing the route for rhizobia internalization. SIMK could eventually play a role in the regulation of receptors for rhizobial extracellular components such as secreted proteins, important for bacterial attachment to the root hair surface. One rhizobial secreted protein, rhicadhesin, was shown to be involved in the attachment of *Rhizobium leguminosarum* to legume root hairs (Smit *et al.*, 1992) at alkaline pH and in a calcium-dependent manner (Laus *et al.*, 2006; Downie, 2010). These hypothetical suggestions will need in-depth experimental investigation to address them in alfalfa–*E. meliloti* early interaction steps. Activation of signalling pathways in the epidermal cells leads to localized inhibition of the tip growth of root hairs and induces their curling, followed by the formation of infection pockets and ITs (Brewin, 2004; Gage 2004). In contrast to the overexpression GFP-SIMK line, where activated SIMK was strongly accumulated around infection pockets and ITs, the transgenic *SIMKK-RNAi* line showed much decreased accumulation. Indeed, the number of formed ITs was significantly lower in the transgenic *SIMKK-RNAi* line, indicating the importance of activated SIMK in infection pockets, which is further required for proper IT formation. Therefore, SIMK down-regulation negatively affects nodule formation, while SIMK overexpression enhances formation of infection pockets and ITs.

In conclusion, we show that active SIMK is associated with *E. meliloti* internalization sites in root hairs and with ITs delivering *E. meliloti* to internal root tissues. SIMK down-regulation negatively affects formation of infection pockets and ITs. The subcellular localization pattern of GFP–SIMK in living cells supported by the immunolocalization pattern clearly demonstrates that active SIMK might be a key player responsible for fine-tuning of the nodulation process in alfalfa. SIMK, therefore, represents a potentially new regulatory protein required for the establishment of efficient symbiotic interaction in alfalfa.

## Supplementary data

The following supplementary data are available at *JXB* online.

Fig. S1. Sample preparation and mounting for LSFM imaging.

Fig. S2. Visualization of GFP–SIMK in uninfected alfalfa root hairs touching the surface of solid FAH-N_2_ medium.

Fig. S3. Quantitative co-localization analysis of MAPKs around infection pockets in root hairs of control and transgenic plants during early stages of *M. sativa*–*E. meliloti* symbiotic interaction.

Fig. S4. Quantitative co-localization analysis of MAPKs around ITs in root hairs of control and transgenic plants during *M. sativa*–*E. meliloti* symbiotic interaction.

Fig. S5. Subcellular immunolocalization of SIMK and activated MAPKs in alfalfa uninfected root hairs with terminated tip growth.

Fig. S6. IT formation in control and transgenic plants after inoculation with *E. meliloti* 10 dpi.

Fig. S7. Effectivity of IT formation in control and transgenic plants after inoculation with *E. meliloti*.

Video S1. 3D volumetric root rendering of the GFP-SIMK line symbiotically interacting with *E. meliloti* expressing mRFP.

Video S2. Orthogonal projection of the root hair showing GFP–SIMK association with rhizobia at the docking site from an *X–Z* view.

Video S3. Orthogonal projection of the root hair showing GFP–SIMK association with rhizobia at the docking site from a *Y–Z* view.

Video S4. Orthogonal projection of the root hair showing GFP–SIMK association with a cluster of rhizobia located at the infection site in the neck of a root hair curl from an *X–Z* view.

Video S5. Orthogonal projection of the root hair showing GFP–SIMK association with a cluster of rhizobia located at the infection site in the neck of a root hair curl from a *Y–Z* view.

Video S6. Orthogonal projection of the root hair showing a very tight association of GFP–SIMK with rhizobia at the infection site before rhizobia entry from an *X–Z* view.

Video S7. Orthogonal projection of the root hair showing a very tight association of GFP–SIMK with rhizobia at the infection site before rhizobia entry from a *Y–Z* view.

Video S8. Orthogonal projection of the root hair showing association of GFP–SIMK with rhizobia entrapped inside a root hair curl at the beginning of infection pocket formation from an *X–Z* view.

Video S9. Orthogonal projection of the root hair showing association of GFP–SIMK with rhizobia entrapped inside a root hair curl at the beginning of infection pocket formation from a *Y–Z* view.

Video S10. Orthogonal projection of the root hair showing association of GFP–SIMK with rhizobia forming colonies within an infection pocket at the beginning of IT formation from a *X–Z* view.

Video S11. Orthogonal projection of the root hair showing association of GFP–SIMK with rhizobia forming colonies within an infection pocket at the beginning of IT formation from a *Y–Z* view.

Video S12. Time-lapse imaging of GFP–SIMK accumulation in the nucleus and at the infection site in the root hair during rhizobia attachment.

Video S13. Time-lapse imaging of GFP–SIMK accumulation around infection pockets in the root hair.

Video S14. Time-lapse imaging of GFP–SIMK accumulation in the nucleus and at the infection site in the root hair during rhizobia attachment analysed by semi-quantitative GFP–SIMK fluorescence intensity distribution.

Video S15. Time-lapse imaging of GFP–SIMK accumulation around infection pockets in the root hair analysed by semi-quantitative GFP–SIMK fluorescence intensity distribution.

erad111_suppl_Supplementary_Figures_S1-S7

erad111_suppl_Supplementary_Video_S1

erad111_suppl_Supplementary_Video_S2

erad111_suppl_Supplementary_Video_S3

erad111_suppl_Supplementary_Video_S4

erad111_suppl_Supplementary_Video_S5

erad111_suppl_Supplementary_Video_S6

erad111_suppl_Supplementary_Video_S7

erad111_suppl_Supplementary_Video_S8

erad111_suppl_Supplementary_Video_S9

erad111_suppl_Supplementary_Video_S10

erad111_suppl_Supplementary_Video_S11

erad111_suppl_Supplementary_Video_S12

erad111_suppl_Supplementary_Video_S13

erad111_suppl_Supplementary_Video_S14

erad111_suppl_Supplementary_Video_S15

## Data Availability

Data that support the findings of this study are available from the corresponding author upon reasonable request.
