# Supplementary material for: Advanced microscopy resolves dynamic localization patterns of stress-induced mitogen-activated protein kinase (SIMK) during alfalfa root hair interactions with Ensifer meliloti
Source: J Exp Bot. 2023 Mar 23;74(12):3729–48. doi: 10.1093/jxb/erad111 (PMC10299790; doi:10.1093/jxb/erad111)
Supplement: erad111_suppl_Supplementary_Figures_S1-S7 [file erad111_suppl_supplementary_figures_s1-s7.pdf]

Sample mounting for LSFM

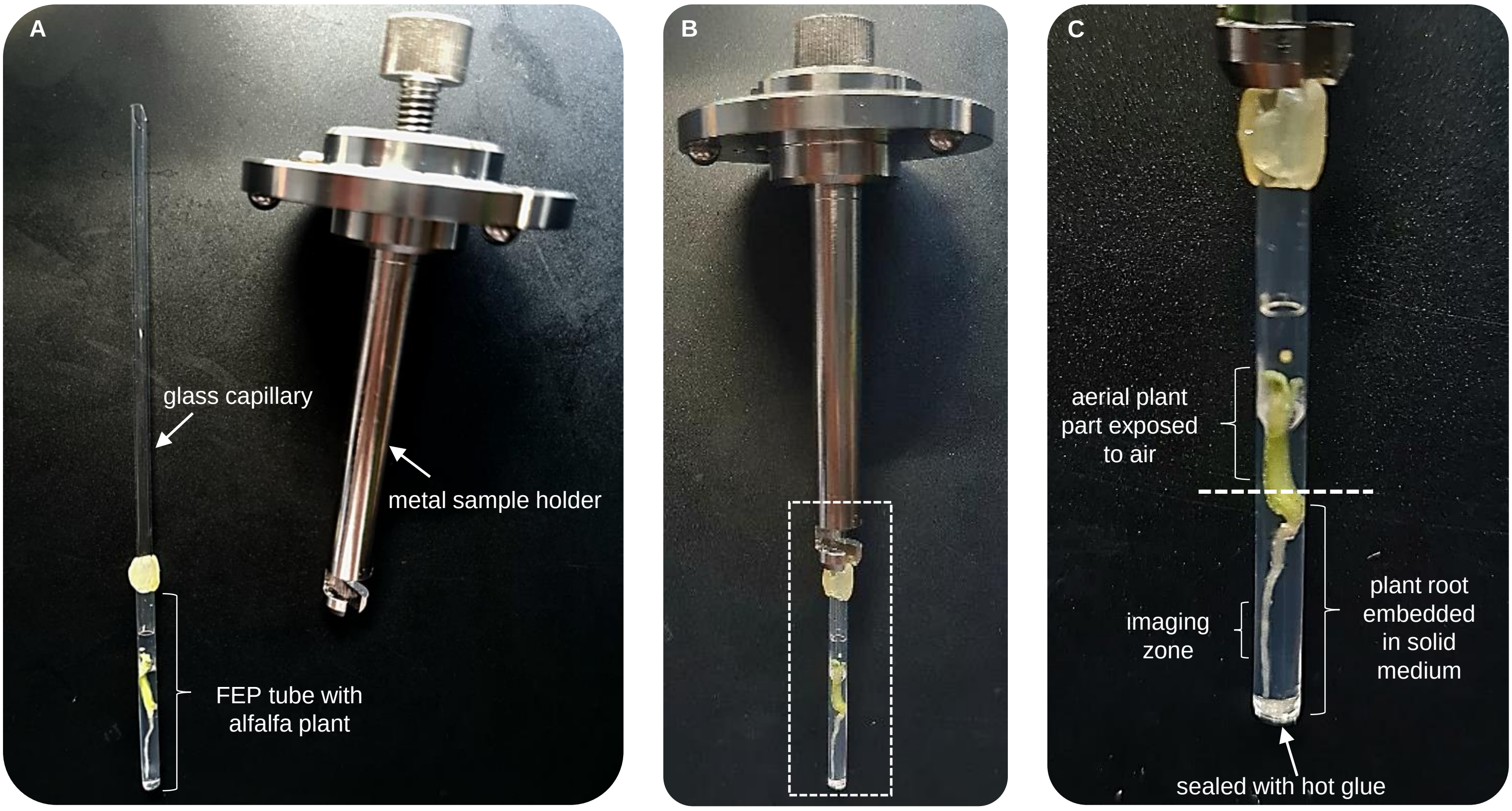

Supplementary Figure S1. Sample preparation and mounting for LSFM imaging. (A-C) Plants of GFP-SIMK line reaching the size of 1.5 cm, growing on solid FAH-N_2 medium and inoculated with mRFP-labelled *E. meliloti* were mounted into the FEP tube connected to the glass capillary with the hot glue **(A)**, and fixed into the standard sample holder **(B)**. White dashed box in **(B)** represents mounted alfalfa plant prepared for imaging, with the root embedded in solid medium and aerial part exposed to air **(C)**. The zone of imaging **(C)** was selected directly in the light-sheet microscope.

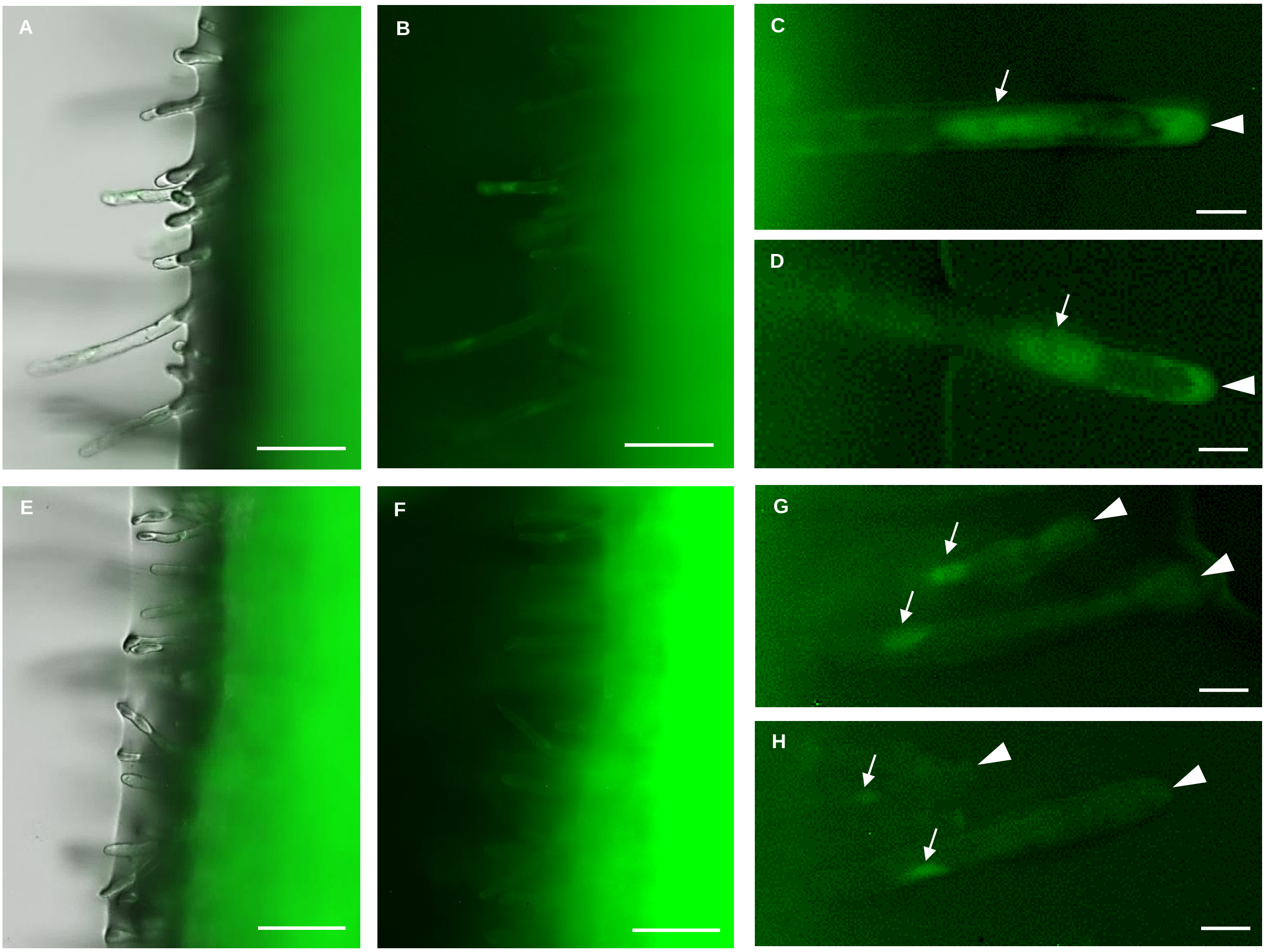

Supplementary Figure S2. Visualization of GFP-SIMK in uninfected alfalfa root hairs touching the surface of solid FAH-N_2 medium. **(A-D)** Growing and **(E-H)** mature non-growing root hairs of alfalfa transgenic GFP-SIMK line. **(A)** Bright-field image overlaid with GFP showing alfalfa root hairs growing on the surface of agar plates as demonstrated by the layer of water formed along the root. **(B)** Accumulation of GFP-SIMK in the apical part of growing root hairs with nuclei positioned close to the root hair tip as shown in detail in **(C,D)**. **(E)** Bright-field image overlaid with GFP showing root hairs of GFP-SIMK line with terminated tip growth. **(F)** In non-growing root hairs, GFP-SIMK is no longer accumulated in root hair tips and nuclei are located near the root hair base as shown in detail in **(G,H)**. Imaging was performed with Axio Zoom.V16 Stereo Zoom microscope (Carl Zeiss, Germany). White arrowheads show root hair tips and arrows represent nuclei inside root hairs. Scale bars = 100 µm **(A,B,E,F)** and 25 µm **(C,D,G,H)**.

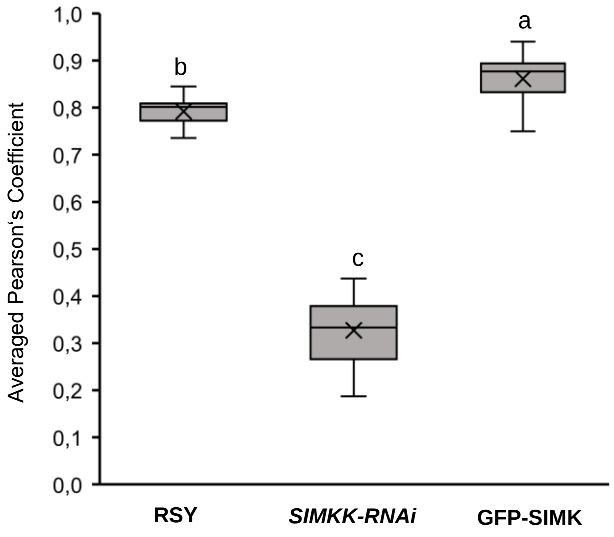

Supplementary Figure S3. Quantitative colocalization analysis of MAPKs around infection pockets in root hairs of control and transgenic plants during early stages of *M. sativa* – *E. meliloti* symbiotic interaction. Averaged Pearson‘s coefficient of quantitative colocalization between SIMK and phosphorylated MAPKs around infection pockets in alfalfa RSY root hairs (N=13 ROIs) and root hairs of transgenic *SIMKK-RNAi* (N=17 ROIs) and GFP-SIMK (N=12 ROIs) plants. Box plots display the first and third quartiles, split by the median; the crosses indicate the mean values; whiskers extend to include the max/min values. Different lowercase letters indicate statistical significance between lines according to one-way ANOVA with post-hoc Tukey HSD test ($P < 0.05$). Error bars show ±SD.

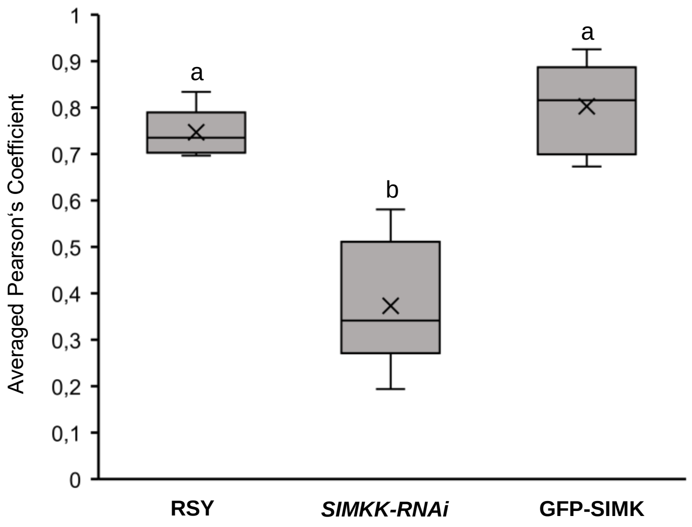

Supplementary Figure S4. Quantitative colocalization analysis of MAPKs around ITs in root hairs of control and transgenic plants during *M. sativa* – *E. meliloti* symbiotic interaction. Averaged Pearson's coefficient of quantitative colocalization between SIMK and phosphorylated MAPKs around ITs in alfalfa RSY root hairs (N=8 ROIs) and root hairs of transgenic *SIMKK-RNAi* (N=16 ROIs) and GFP-SIMK (N=8 ROIs) plants. Box plots display the first and third quartiles, split by the median; the crosses indicate the mean values; whiskers extend to include the max/min values. Different lowercase letters indicate statistical significance between lines according to one-way ANOVA with post-hoc Tukey HSD test ($P < 0.05$). Error bars show ±SD.

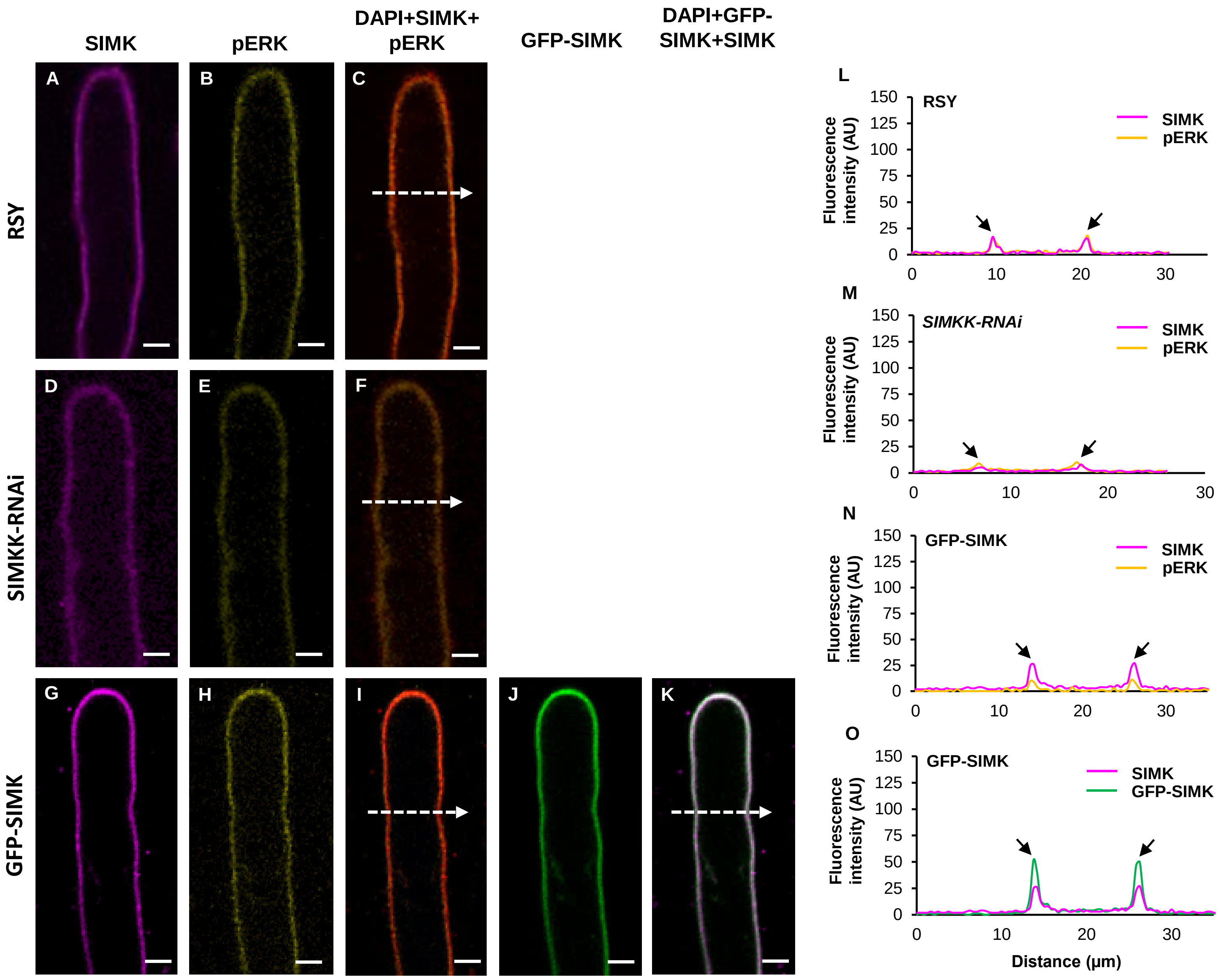

Supplementary Figure S5. Subcellular immunolocalization of SIMK and activated MAPKs in alfalfa uninfected root hairs with terminated tip growth. (A,D,G) SIMK immunostained with SIMK-specific antibody in RSY **(A)**, *SIMKK-RNAi* **(D)** and GFP-SIMK **(G)** lines. **(B,E,H)** Activated MAPKs immunostained with phospho-specific pERK 44/42 antibody in RSY **(B)**, *SIMKK-RNAi* **(E)** and GFP-SIMK **(H)** lines. **(C,F,I)** Overlay of SIMK and activated MAPKs in RSY **(C)**, *SIMKK-RNAi* **(F)** and GFP-SIMK **(I)** plants. **(J,K)** GFP-tagged SIMK overlaid with DAPI **(J)** and overlay of GFP-tagged SIMK and SIMK immunostained with SIMK-specific antibody in transgenic GFP-SIMK line **(K)**. **(L,M,N,O)** The fluorescence intensity distribution of SIMK, activated MAPKs and GFP-tagged SIMK was measured along profiles indicated by white dashed arrows in **(C,F,I,K)**. Black arrows indicate the plasma membrane of root hair. Scale bar = 5 μm **(A-K)**.

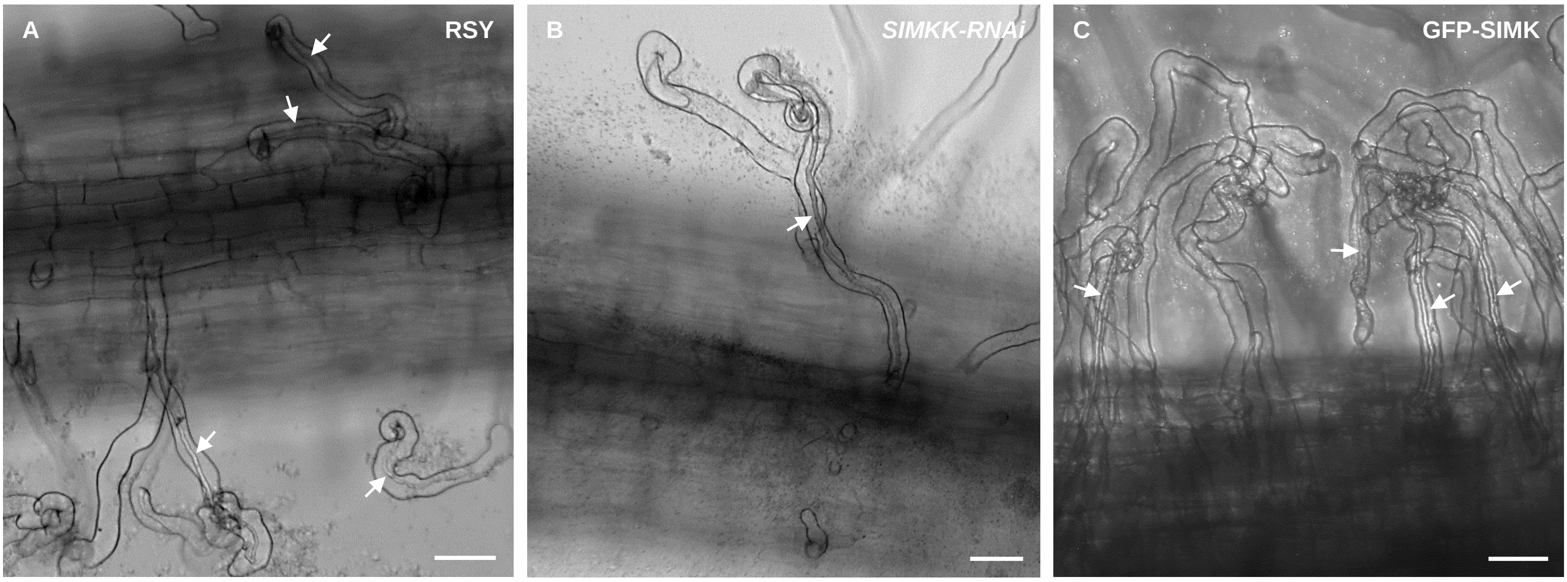

Supplementary Figure S6. ITs formation in control and transgenic plants after inoculation with *E. meliloti* 10 dpi. Representative images of ITs formed inside root hairs of control RSY **(A)** and transgenic *SIMKK-RNAi* **(B)** and GFP-SIMK **(C)** lines 10 dpi with *E. meliloti* wild-type. White arrows show individual ITs inside root hairs. Scale bar = 50 µm **(A-C)**.

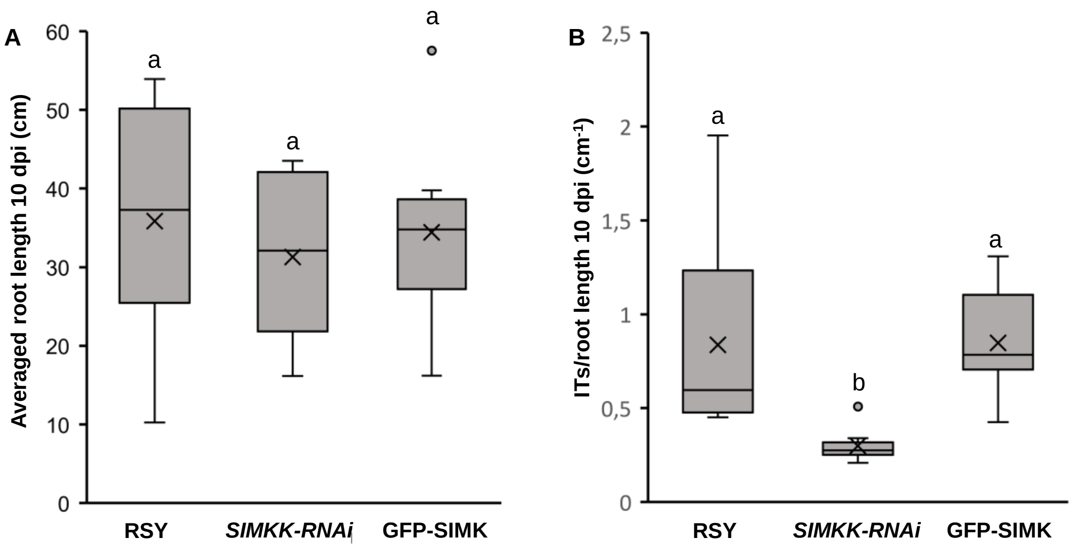

Supplementary Figure S7. Effectivity of ITs formation in control and transgenic plants after inoculation with *E. meliloti*. (A-B) Averaged root length **(A**; N=8 for RSY; N=10 for *SIMKK-RNAi* line; N=8 for GFP-SIMK line) and number of ITs per length of the whole root system **(B)** 10 dpi with *E. meliloti* in alfalfa RSY (N=8) and transgenic *SIMKK-RNAi* (N=10) and GFP-SIMK (N=8) lines. Box plots display the first and third quartiles, split by the median; the crosses indicate the mean values; whiskers extend to include the max/min values. Different lowercase letters indicate statistical significance between lines according to one-way ANOVA with post-hoc Tukey HSD test ($P < 0.05$). Error bars show ±SD.
